# Milk proteins as a feed restriction signature indicating the metabolic adaptation of dairy cows

**DOI:** 10.1038/s41598-022-21804-1

**Published:** 2022-11-07

**Authors:** A. Leduc, S. Le Guillou, L. Bianchi, L. Oliveira Correia, M. Gelé, J. Pires, P. Martin, C. Leroux, F. Le Provost, M. Boutinaud

**Affiliations:** 1 Université Paris-Saclay, INRAE, AgroParisTech, GABI, 78350 Jouy-en-Josas, France; 2 INRAE, Institut Agro Rennes Angers, PEGASE, 35590 Saint-Gilles, France; 3 Institut de L’Elevage, 75012 Paris, France; 4 INRAE, AgroParisTech, Micalis Institute, PAPPSO, Université Paris-Saclay, 78350 Jouy-en-Josas, France; 5 INRAE, UMRH, Vetagro Sup, Université Clermont Auvergne, 63122 Saint-Genès-Champanelle, France

**Keywords:** Biochemistry, Physiology

## Abstract

Milk production in dairy cows is affected by numerous factors, including diet. Feed restriction is known to have little impact on milk total protein content but its effect on the fine protein composition is still poorly documented. The objective of this study was to describe the effects of two feed restriction trials of different intensities on the milk protein composition of Holstein cows. One restriction trial was of high intensity (H: 8 mid-lactation Holstein cows) and the second of moderate intensity (M: 19 peak lactation Holstein cows). Feed restriction decreased the milk protein yield for caseins under the M trial and of all six major milk proteins under the H trial. These decreased yields lead to lower concentrations of αs1-, αs2- and β-caseins during the H trial. The milk proteome, analyzed on 32 milk samples, was affected as a function of restriction intensity. Among the 345 proteins identified eight varied under the M trial and 160 under the H trial. Ontology analyses revealed their implication in carbohydrate, lipid and protein metabolisms as well as in the immune system. These proteins reflected adaptations of the animal and mammary gland physiology to feed restriction and constituted a signature of this change.

## Introduction

Milk is a secretory product rich in proteins, lipids, lactose and minerals, rendering it a unique source of nutrient. Milk yield and composition is influenced by numerous factors such as genetics, environment, health status, lactation stage and nutrition. Undernutrition in dairy cows can rapidly induce a negative energy balance that is known to impact metabolism through body reserve mobilization which might affect health (notably involving an increased risk of ketosis) and also milk production and hence economic outcomes. Such modifications obviously affect milk composition. The extent of feed restriction effects depend on its duration, intensity and the lactation stage at which it occurs^1^.

Feed restriction experiments performed on dairy cows have generally shown little or no effect on milk total protein content, and only a few of these studies explored their effect on fine protein composition. Bovine milk contains six major proteins: four caseins (CN) that account for about 80% of total proteins (αs1-, αs2-, β- and κ-CN) and two major whey proteins: α-lactalbumin (α-LA) and β-lactoglobulin (β-LG). Some feed restriction studies focused their analyses on these six proteins, such as that by Gellrich et al.^2^ who did not observe any variations in concentration during a 3 day feed restriction period of moderate intensity in early and mid-lactation. Similarly, Vanbergue et al.^3^ did not see any variations in major milk protein concentrations after 21 days of feed restriction of moderate intensity (− 25% of dry matter intake (DMI)) but showed that αs2- and β-CN concentrations in milk were lower when cows were fed with a conserved grass diet than when they were fed with a corn silage diet, highlighting the effect of energy and protein intake on the major milk protein profile. However, Auldist et al.^4^ described decreased concentrations of every CN and β-LG during an 8 day feed restriction of high intensity based on pasture allowance (estimated at > 45 *versus* 18 kg DM/day per cow). Based on these data, the intensity of the feed restriction may affect major milk protein concentrations. However, milk does not solely contain these six proteins, and a few proteomic analyses have reported on the effects of feed restriction on global milk protein profiles. Only one studied proteome variations induced by diet; it involved different ratios of dietary rumen degradable protein to rumen undegradable protein, and the authors did not observe any effects on low-abundance proteins^5^. Furthermore, an aggregation of proteomic data on cow’s milk – which included 20 publications – reported a total of 4,654 unique proteins^6^. These proteins varied throughout different lactation stages and originated from various tissues such as the liver, adipose tissue or mammary gland; they may therefore have reflected mammary gland metabolism or even global metabolism at a given time. The authors suggested that some of the milk proteins detected exclusively during early lactation might be biomarkers of a negative energy balance^6^.

The aim of the present study was to describe the effects of feed restrictions of different intensity on milk protein composition in dairy cows in order to identify proteins that might characterize this physiological stress. Two feed restriction trials were applied, one of high intensity (H) and the other of moderate intensity (M). Milk sampled before, during and after these restriction periods was used to explore its major protein profiles and proteomes.

## Methods

### Animals, experimental designs and sampling

This article reports on the results of two distinct feed restriction trials: one of high intensity (H) and the other of moderate intensity (M).

The H trial was conducted at the INRAE Herbipôle experimental farm (UE Herbipôle, 15,190 Marcenat, France; https://doi.org/10.15454/1.5572318050509348E12). All procedures involving animals were approved by the local Ethics Committee of the Auvergne-Rhône-Alpes region and the French Ministry of Higher Education, Research and Innovation (APAFIS #3737–2015043014541577v2).

**Figure 1:**
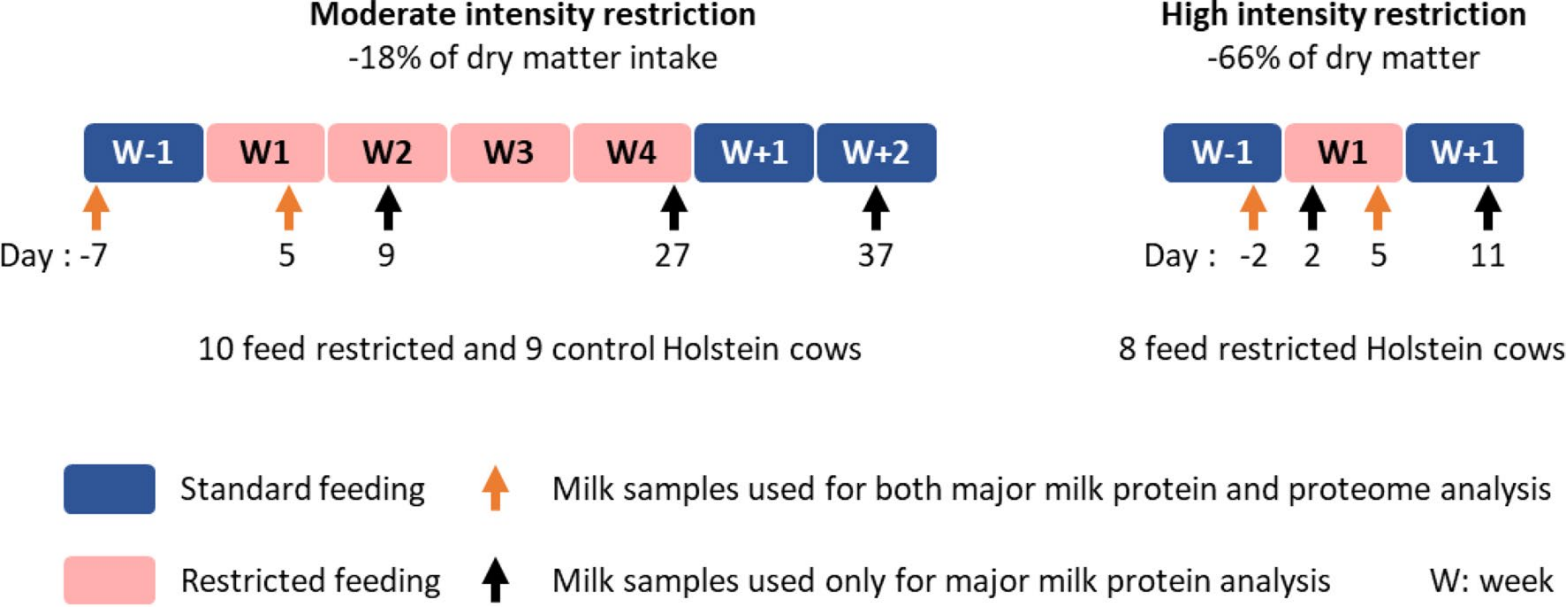
Experimental design of the two feed restriction trials: one of moderate intensity (M) and one of high intensity (H) in dairy cows. Before and after the feed restriction periods, all cows received 100% of their ad libitum dry matter intake. During feed restriction, rations were reduced relative to the pre-experimental period, except for control cows in the M trial.

Eight multiparous mid-lactation (165 ± 21 days in milk (DIM); lactation ranks 2 to 5) Holstein cows were used to study the effects of an intense feed restriction designed to reduce their net energy for lactation (NE_L_) by 50%, as described by Billa et al.^7^. The experiment was divided into three periods: pre-restriction (day (d) -3 to -1), restriction (d 1 to 6) and post-restriction (d 7 to 18). During the pre- and post-restriction periods, the cows were fed ad libitum with a total mixed ration. During the restriction period, the feed allowance was reduced to 50% of individual NE_L_ requirements calculated from body weight, DMI and milk yield and composition, as recorded during the pre-restriction period. Milk samples were collected during morning milking, before feed distribution, on d-2, 2, 5 and 11 relative to the initiation of feed restriction (Fig. 1).

The M trial was performed at the INRAE PEGASE experimental farm (IEPL, 35,650 Le Rheu, France; https://doi.org/10.15454/yk9q-pf68). All procedures involving animals were approved by the local Ethics Committee in Animal Experiment of Rennes and the French Ministry of Higher Education, Research and Innovation (APAFIS #3063–2,015,110,215,066,393).

Nineteen peak lactation (77 ± 5 DIM; lactation ranks 1 to 4) Holstein cows were used to study the effects of a moderate feed restriction designed to reduce their DMI by 20%, as described by Herve et al.^8^. The experiment was divided into three periods: pre-restriction (d -20 to -1), restriction (d 1 to 29) and post-restriction (d 30 to 67). During the pre- and post-restriction periods the cows were fed ad libitum with a total mixed ration. After the pre-restriction period, the cows were assigned to either a control group (n = 9) or a feed-restricted group (n = 10) based on pre-restriction DMI, lactation rank, DIM, milk yield and composition. During the restriction period, control cows were fed 100% of their ad libitum DMI whereas feed-restricted cows were fed at 80% of their ad libitum DMI, as recorded during the pre-restriction period. Milk samples were collected during morning milking, before feed distribution, on d-7, 5, 9, 27 and 37 relative to the initiation of feed restriction (Fig. 1).

### Profiling of major milk proteins

All milk samples collected before, during and after feed restriction under both trials were used to profile major milk proteins. Milk samples that had been stored at -80 °C were thawed for 4 h at 4 °C and then centrifuged for 20 min at 2600 g and 4 °C. The fat supernatant was then removed with a spatula. Skim milk proteins were separated by reverse-phase (RP) HPLC using an Ultimate LC 3000 system (Thermo Fisher Scientific, Waltham, MA) as described by Fang et al.^9^. The relative concentrations of the six major milk proteins (αS1-CN, αS2-CN, β-CN, κ-CN, α-LA, and β-LG) were estimated by the integration of peaks from UV Absorbance recorded at 214 nm, as a percentage of the total area of peaks, for each individual milk sample. Protein concentrations (g/kg) and yields (g/d) were then calculated from their relative abundance, total protein content and milk yield, all measured at the same sampling date.

### Proteomic profiling

Samples from eight restricted cows under each trial, collected on the sampling day before feed restriction and at day 5 during feed restriction were used for proteomic profiling. For the proteome analysis, samples of 15 μL skimmed milk containing around 30 g/L proteins, were loaded into 1D gel electrophoresis (NuPAGE 4–12% Bis–Tris Gel). After the excision of gel bands, the proteins were reduced (DTT, Sigma), alkylated (iodoacetamide, Sigma) and digested with 1 µg trypsin. The peptides were desalted on a Strata-X column (33 µm, 30 mg, Phenomenex), dried under a vacuum and taken up in 30 μL loading buffer (0.08% trifluoroacetic acid, 2% acetonitrile) for LC–MS/MS proteome analysis.

Four μl of each sample were injected into an UltiMate 3000 RSLCnano System (Thermo Fisher Scientific) coupled to an Orbitrap Fusion Lumos Tribrid (Thermo Fisher Scientific). Separation was performed at a flow rate of 0.3 μL/min with a linear gradient of 6–30% (0.1% formic acid, 80% acetonitrile) for 110 min, 30–98% for 10 min and 98% for 10 min. A complete run, including regeneration with 99% buffer (0.1% formic acid, 2% acetonitrile) required 147 min. Nanospray ionization was performed by applying 1.6 kV in a positive mode. Capillary transfer was performed at 275 °C using a capillary probe SilicaTip Emitter 10 μm.

The mass spectrometer was operated in data dependent acquisition mode. Full MS scans were captured in the Orbitrap (scan range 400–1500 m/z) with a resolution of 120,000. Dynamic exclusion was set at 10 ppm with a duration of 80 s, and the intensity threshold was fixed at 5 × 10^4^. MS2 was performed using High Collision Dissociation (HCD) in the Orbitrap at a resolution of 30,000. Polysilaxolane ions m/z 445.12002, 519.13882, 593.15761, and 667.1764 were used for internal calibration.

Protein identification was performed using X!TandemPipeline C +  + 0.4.17^10^ and the *Bos taurus* UniProtKB database (version 2019, 46,697 entries). Data filtering was achieved according to a peptide E-value < 0.01, protein log (E-value) <  − 4 and a minimum of two identified peptides per protein. The peptide and protein False Discovery Rates (FDR) were estimated at 0.68% and 0.25%, respectively. MS1 peaks were detected and aligned using MassChroQ 2.2.12^11^.

The relative quantification of protein abundances was performed using two complementary methods: spectral counting (SC) defined as the number of MS2 spectra assigned to a protein^12^, and extracted ion chromatograms (XIC) defined as the sum of the MS1 intensities of all peptides associated with a protein. The XIC method is suited to detecting subtle differences in protein abundance based on specific peptide data, while SC only enables the detection of larger abundance variations, including that of presence/absence.

### Statistical analyses

Statistical analyses were performed using R software v4.0.2 (R Core Team, 2020, http://www.R-project.org) with the lme4 package version 1.1–23. Analyses of variance of the major milk protein data were performed using a mixed model that included day, diet and their interaction as fixed effects, and the cow as a random effect. The restriction effect was calculated by comparison with pre-restriction values except for RP-HPLC analysis on M trial, where pre-restriction values were used as co-factors and feed restriction effects were calculated by comparison with the control group. A trial effect was analyzed for pre-restriction data using a linear model. Analyses of variance of the proteomic data were performed using a mixed model that included diet as a fixed effect and the cow as a random effect. Feed restriction effects were calculated by comparison with pre-restriction values. The threshold for statistical significance was set at *P* < 0.05 and trend-level significance was defined as 0.05 ≤ *P* < 0.10. Moreover, for statistical analysis of proteomic data, proteins showing numbers of spectra lower than 5 in all the samples or fold change above or below 1.5 were removed.

### Exploration of in silico metabolic pathways

Enrichment analyses were carried out using the PANTHER overrepresentation test^13^ with the GO ontology database (https://doi.org/10.5281/zenodo.5228828; Released 2021–08-18). The *Bos taurus* database (22,798 proteins) was used as the reference list and Fisher’s test was performed with a false discovery rate (FDR) cut-off point set at 0.05. Hierarchy sorting was used to identify families of gene ontology (GO) terms.

## Ethics declaration

All the experimental procedures were carried entirely under animal welfare guidelines (including ARRIVE guidelines) and were approved by the local Ethics Commitees in Animal Experiment and the French Ministry of higher Education, Research and Innovation.

## Results

### Effects of restrictions on animal performance

The results concerning DMI, energy balance, milk yield and composition and plasma NEFA from both trials have been reported previously^7,8^. The two feed restriction protocols were of different intensities as indicated by the reduction in DMI (-18% vs -66%, respectively, in the M and H trials; Fig. 1, Table 1) and took place at different stages of lactation, the cows involved in the M trial being at peak lactation (77 ± 5 DIM) and those in H trial at mid-lactation (165 ± 21 DIM). These feed restrictions led to a negative energy balance with decreased milk yields (− 12 and − 40%, respectively, in the M and H trials; Table 1) after 5 days of feed restriction. These protocols induced changes to the milk composition with reduced milk lactose content in both trials, reduced protein content only in H trial and no significant effect on fat content (Table 1). These effects were accompanied by body reserve mobilization, as shown by an increase in plasma non-esterified fatty acids^7,8^ (Table 1).

**Table 1:** Effects of feed restriction on dry matter intake (DMI), energy balance (NE_L_), milk yield and composition and plasma non-esterified fatty acid (NEFA) levels before and 5 days (d) after the initiation of the two feed restriction trials: one of moderate intensity (M) and the other of high intensity (H).

	M trial^8^				H trial^7^			
	d-7	d5	SEM	*P*-value	d-2	d5	SEM	*P*-value
DMI (kg/d)	23.4	19.1	0.91	< 0.001	25.9	8.7	0.63	< 0.001
NE_L_ (MJ/d)	− 15.8	− 25.6	3.52	0.023	41.4	− 33.9	5.31	< 0.001
Milk yield (kg/d)	38.5	33.7	2.42	0.058	28.6	17.1	1.54	< 0.001
Milk fat (%)	3.45	3.97	0.176	0.087	3.71	3.77	0.135	0.760
Milk protein (%)	2.85	2.81	0.063	0.481	3.04	2.90	0.045	0.020
Milk lactose (%)	5.04	4.89	0.043	0.024	5.07	4.78	0.068	0.012
Plasma NEFA (μM)	165	524	52	< 0.001	104	696	60	< 0.001

### Variations in major milk proteins induced by feed restriction

**Figure 2:**
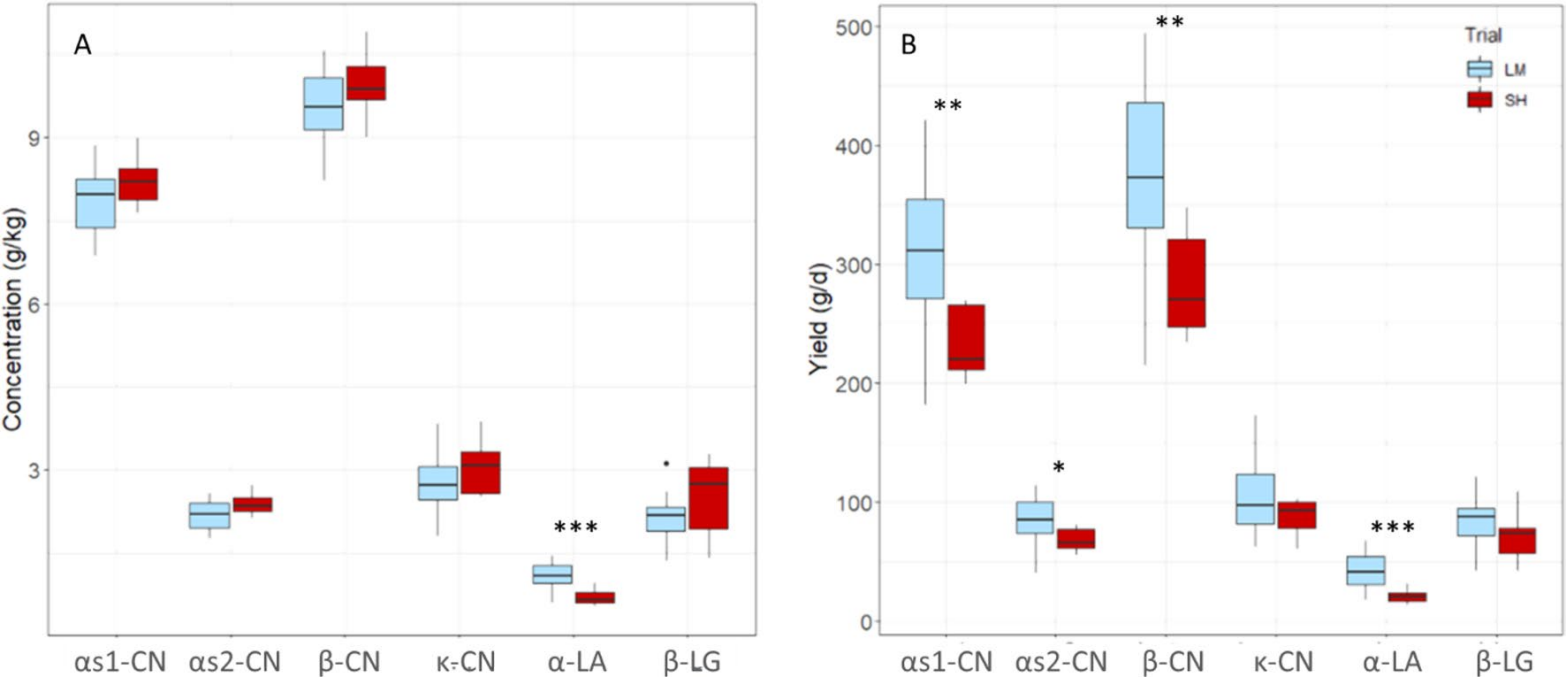
Boxplots showing the comparison of the concentrations (**A**) and yields (**B**) of the six major milk proteins analyzed using RP-HPLC before the feed restriction period of both trials. In light blue the trial of moderate intensity (M) (10 cows), and in red the feed restriction trial high intensity (H) (8 cows). Analyses of variance between the trials: * *P* ≤ 0.05; ** *P* ≤ 0.01; *** *P* ≤ 0.001.

Before studying the effects of feed restriction, a comparison of the skim milk protein composition between both trials was performed before feed restrictions, in order to ensure that the comparison of feed restriction trials was relevant. Comparison of the milk samples collected before the feed restriction period (d-7 and d-2 for M and H, respectively) showed that the concentrations in each major milk protein did not differ between the trials despite the difference in lactation stage, except for α-LA for which concentrations were higher in M samples than in H samples (*P* < 0.001, Fig. 2A). The yields of most major protein were higher in M trial than in H trial, particularly for α-LA, αs1-, αs2- and β-CN (*P* = 0.0004; 0.006; 0.02 and 0.006, respectively, Fig. 2B).

**Figure 3:**
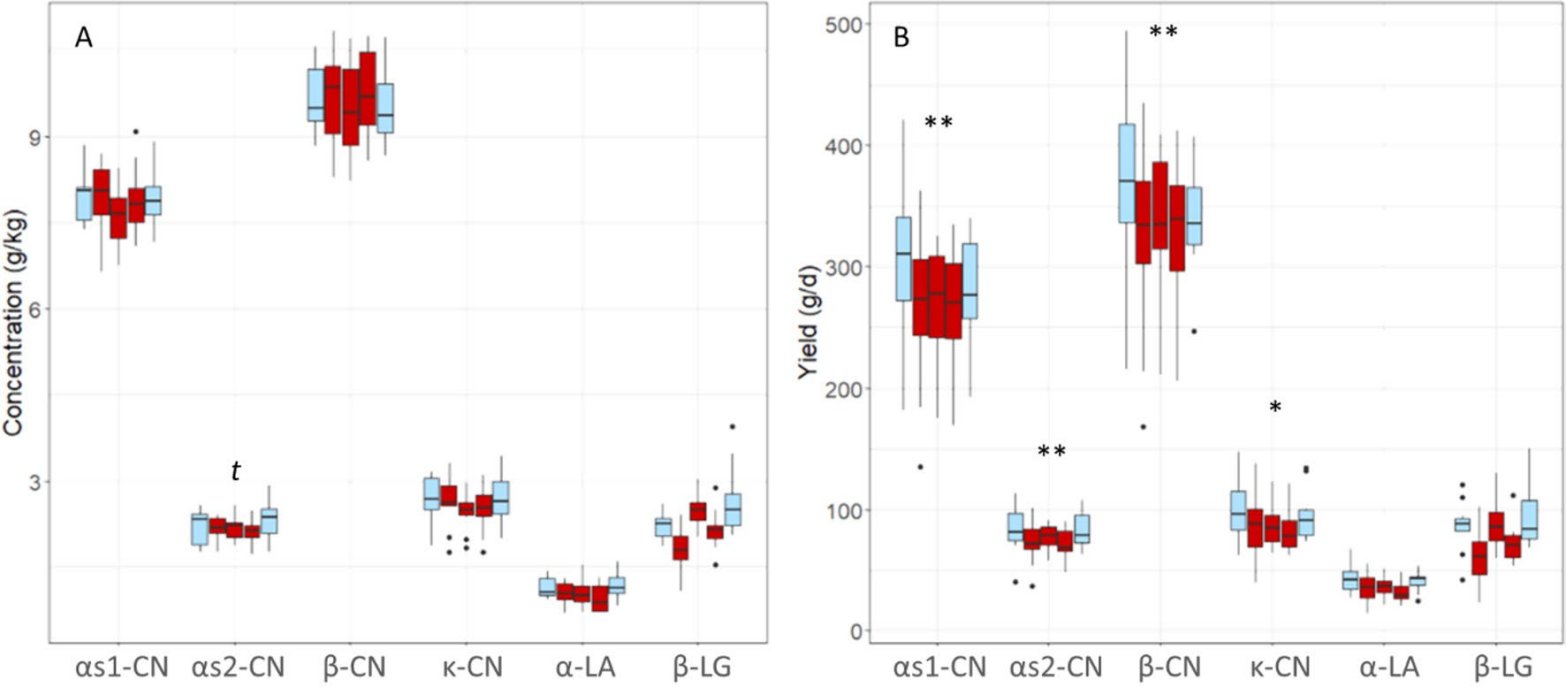
Variations in the concentrations (**A**) and yields (**B**) of the six major milk proteins analyzed using RP-HPLC during the feed restriction trial of moderate intensity (M) (− 18% dry matter intake) in the 10 feed restricted cows. The five boxplots represent each day of sampling for each protein: in light blue during ad libitum feeding (d -7 and 37) and in red during the restriction period (d 5, 9 and 27). Analyses of variance between diets: *t*: *P* ≤ 0.1; * *P* ≤ 0.05; ** *P* ≤ 0.01; *** *P* ≤ 0.001.

In the ten cows involved in the M trial, milk major proteins were analyzed in samples collected seven days before the feed restriction period, on days 5, 9 and 27 after the start of feed restriction and on day 37, after the return to *ad-libitum* feeding. None of the major protein concentrations was significantly affected by the moderate feed restriction except for a tendency towards a lower αs2-CN concentration (*P* = 0.06) during the restriction period. Nevertheless, the yields of αs1-, αs2-, β- and κ-CN decreased under feed restriction (*P* = 0.004; 0.005; 0.002 and 0.05, respectively; Fig. 3). These yields rapidly returned to pre-restriction values after *ad-libitum* refeeding.

**Figure 4:**
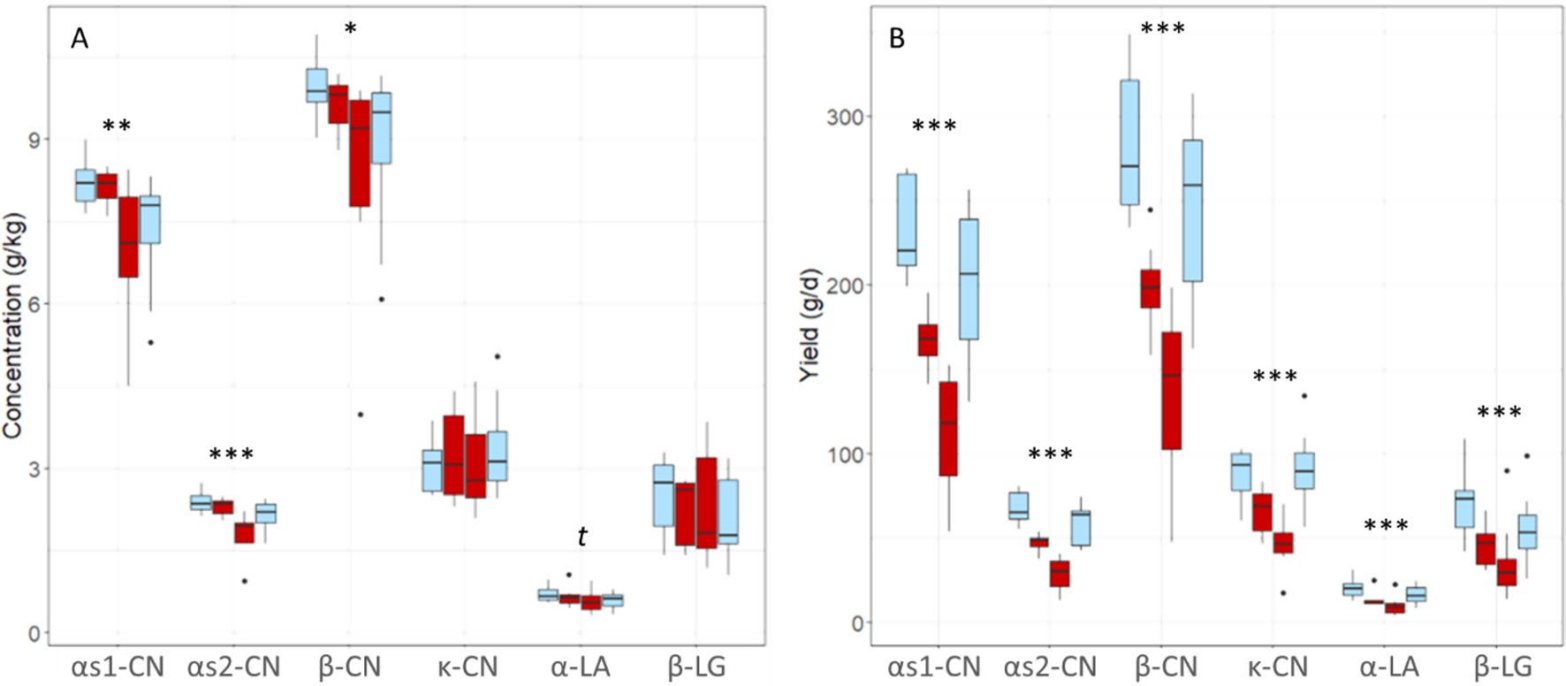
Variations in the concentrations (**A**) and yields (**B**) of the six major milk proteins analyzed using RP-HPLC during the feed restriction trial of high intensity (H) (− 66% dry matter intake) in the eight feed restricted cows. The four boxplots represent each day of sampling for each protein: in light blue during ad libitum feeding (d-2 and 11) and in red during the restriction period (d2 and 5). Analyses of variance between pre-restriction and restriction periods: *t*: *P* ≤ 0.1; * *P* ≤ 0.05; ** *P* ≤ 0.01; *** *P* ≤ 0.001.

In the eight cows involved in the H trial, milk major proteins were analyzed in samples collected two days before the feed restriction period, on days 2 and 5 after the start of feed restriction and on day 11, after the return to *ad-libitum* feeding. αs1-, αs2- and β-CN concentrations decreased significantly under feed restriction during this trial (*P* = 0.004; 0.0004; 0.01, respectively) and α-LA concentrations tended to decrease (*P* = 0.06). The quantities of all six major milk proteins produced per day decreased significantly under the high intensity feed restriction when compared to pre-restriction values (*P* < 0.001; Fig. 4). These concentrations and yields quickly returned to pre-restriction values after *ad-libitum* refeeding.

### Proteome variations induced by feed restriction

**Figure 5:**
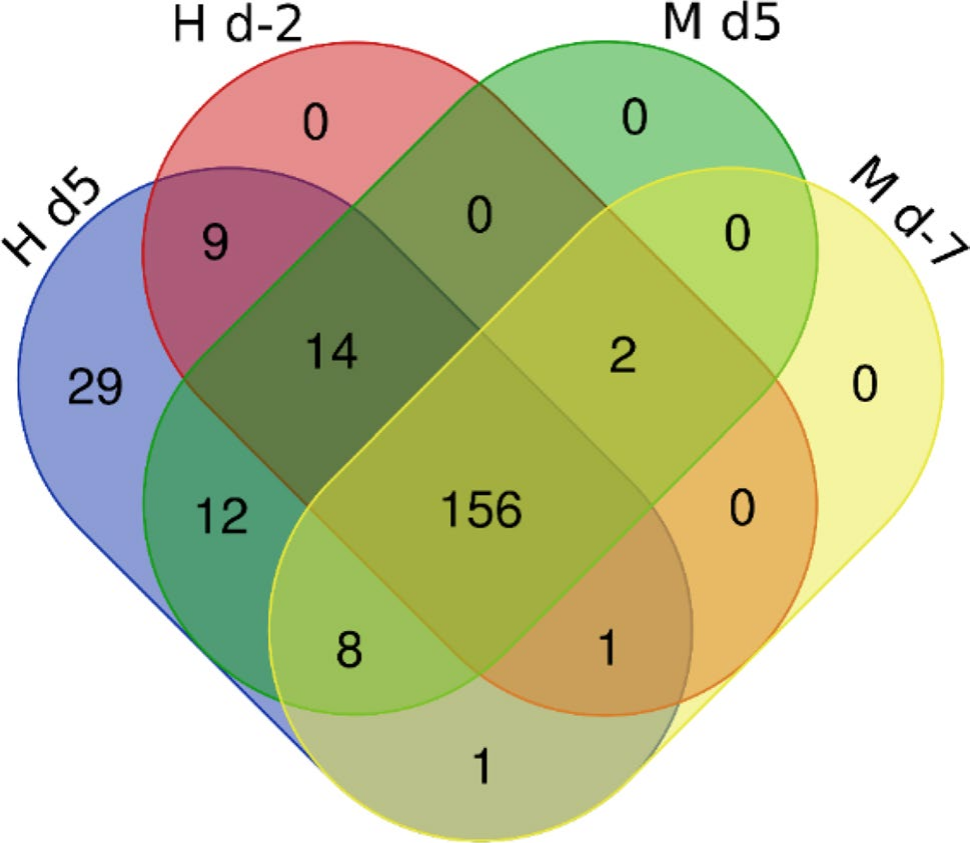
Comparison of milk proteomes from 16 Holstein cows involved in feed restriction trials of moderate (M: -18% of dry matter intake) or high (H: -66% of dry matter intake) intensity, before and during feed restriction periods (d: days relative to the initiation of feed restriction): Venn diagram representing the number of identified proteins after filtration on SC and XIC quantifications.

**Figure 6:**
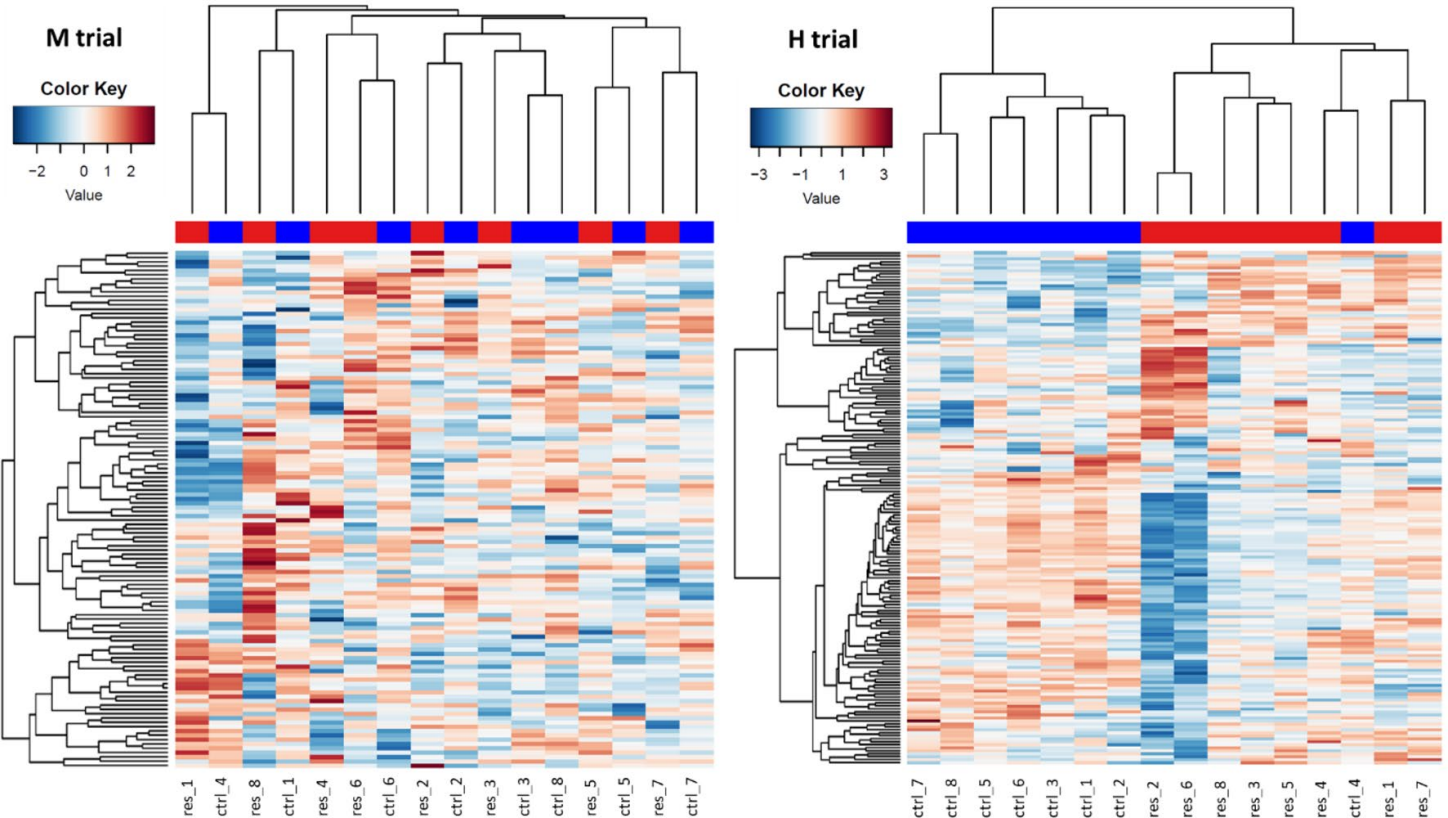
Comparison of individual skim milk proteome before (ctrl, blue) and after 5 days (res, red) of feed restriction in two trials. Heat map of pairwise Pearson’s correlation of the XIC counts for M trial on left side (8 Holstein cows, -18% of dry matter intake) and H trial on right side (8 Holstein cows, − 66% of dry matter intake).

Proteome analyses were performed using LC–MS/MS on 32 skim milk samples, from eight cows in each trial, before and during feed restriction period. Quality of proteomes was assessed by Pearson's correlation between all Normalized Spectral Abundance Factor (NSAF^14^) in paired samples within each trial and feeding condition (average *r* = 0.97). This enabled the identification of 345 different proteins in these skim milk samples (Supplementary Data S1. A). Filters were applied to retain peptides with E-value < 0.01, proteins with log (E-value) <  − 4 and a minimum of two identified peptides per protein. This filtering left 151 and 152 significantly abundant proteins respectively for SC and XIC quantifications, for a total of 232 unique proteins (Supplementary Data S1.B). Among them 52 were found in every milk sample and 156 were found in at least one sample from each trial and diet. 191 of the 232 identified protein were found before the feed restriction period, with 23 proteins exclusive to H trial and nine exclusive to M trial during this period (Fig. 5). All 232 proteins were found in H milk samples, 50 of them were only found during the feed restriction and two of them were only found before the feed restriction period. 194 proteins were found during M trial, 26 of them were only found during feed the restriction and 2 before the feed restriction period (Fig. 5). The differences of milk protein abundances led to a good discrimination between feed conditions for H trial whereas individual variations had more influence during M trial (Fig. 6). The mass spectrometry proteomics data have been deposited to the ProteomeXchange Consortium via the PRIDE partner repository with the dataset identifier PXD033969^15^.

**Figure 7:**
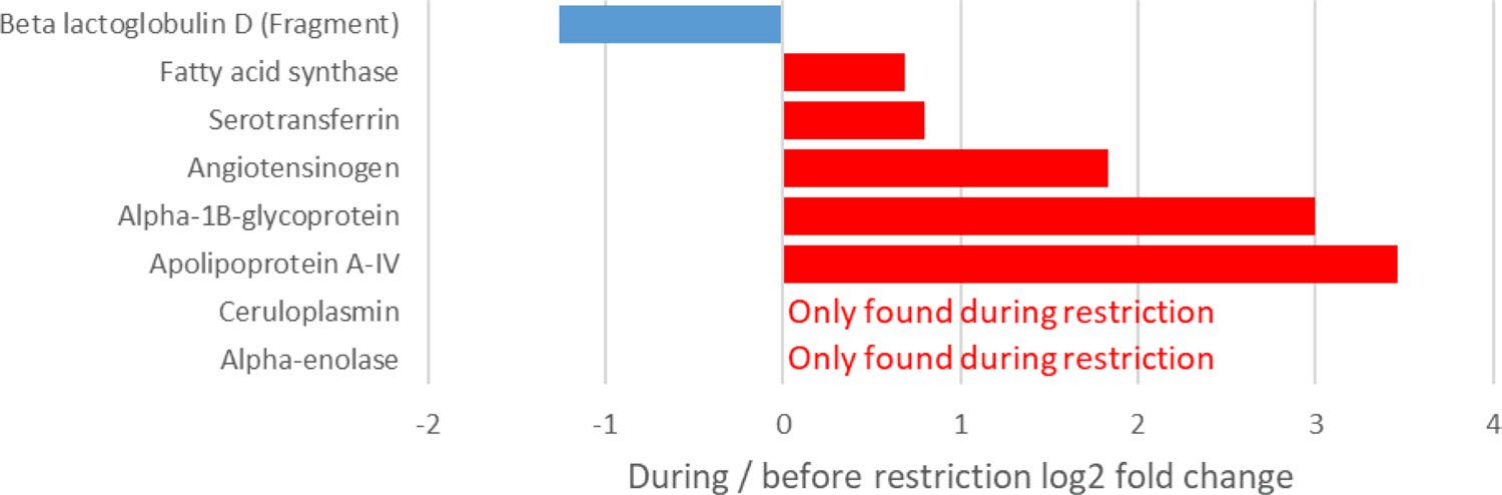
Variations in protein abundance in milk from eight dairy cows after five days of moderate feed restriction (M trial: − 18% of dry matter intake). Analyses of variance between the pre-restriction and restriction periods: *P* ≤ 0.05.

In the M trial, statistical analyses led to the identification of eight proteins whose abundance varied significantly during feed restriction (*P* < 0.05; fold change > 1.5). In particular, the abundance of beta lactoglobulin D decreased whereas those of apolipoprotein A-IV, alpha-1B-glycoprotein, angiotensinogen, serotransferrin and fatty acid synthase increased under feed restriction. In addition, two of the 26 proteins that were only detected in the milk during feed restriction were confirmed as significantly variable: Alpha-enolase and ceruloplasmin (Fig. 7).

**Figure 8:**
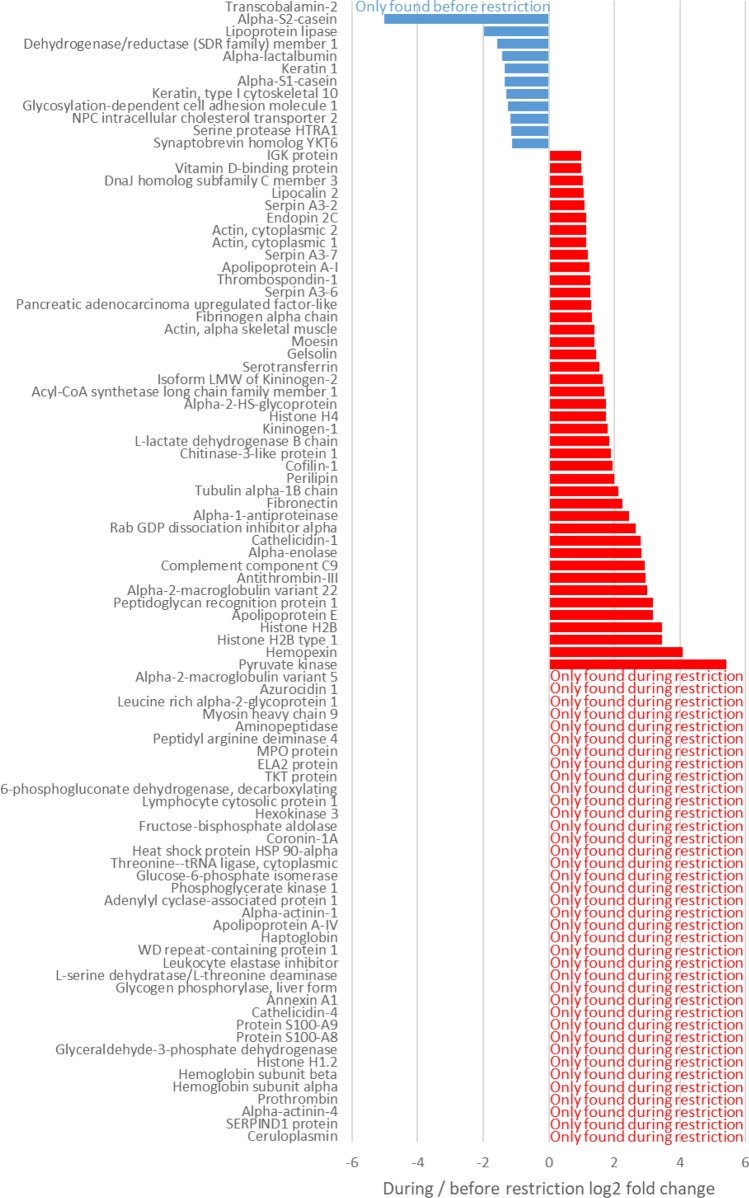
Proteins identified in milk from eight cows subjected to high intensity feed restriction (H trial: − 66% dry matter intake) with a log2 fold change in abundance > 1 or <  − 1 after five days of feed restriction during the H trial. Analyses of variance between pre-restriction and restriction periods using a *P* value ≤ 0.05.

In the H trial, proteome analysis led to the identification of 160 proteins whose abundance varied during feed restriction (*P* < 0.05; fold change > 1.5), including 92 with a log2 fold change > 1 or <  − 1 (Fig. 8; Supplementary Data S2). Among these 160 proteins, 43 of the 50 proteins that were only present in milk during feed restriction were confirmed as significantly variable and Transcobalamin-2 was confirmed as only present before restriction. The abundance of 39 proteins decreased and that of 77 increased during feed restriction.

Seven of the eight differentially abundant proteins in the restriction period of the M trial were in common with the differentially abundant proteins found during the restriction period of the H trial. The one that was exclusive of H trial during restriction was a fragment of β-LG D, a rare genetic variant.

### Biological processes affected by feed restriction

Gene Ontology (GO) analyses were only performed on data from the H trial because too few proteins varied under feed restriction during the M trial to perform a relevant GO analysis. The GO analyses of the H trial showed that the major cellular processes affected by feed restriction were related to carbohydrate, protein and lipid metabolisms as well as immune system processes (Table 2).

**Table 2:** Milk proteins affected by feed restriction during the H trial (short duration and high intensity: − 66% of DMI for 6 days) (n = 8 Holstein dairy cows) and involved in GO terms related to carbohydrate (A), lipid (B), and protein (C) metabolisms or the immune system (D). Fold change (FC) is the log2 ratio between d 5 and d-2. ∞ represent proteins only found in milk during feed restriction.

Protein name	Gene symbol	log2 FC	adj p.value
**A. Carbohydrate metabolism**			
6-phosphogluconate dehydrogenase, decarboxylating	PGD	∞	9.62E-05
Fructose-bisphosphate aldolase	ALDOA	∞	1.44E-05
Glucose-6-phosphate isomerase	GPI	∞	1.86E-04
Glyceraldehyde-3-phosphate dehydrogenase	GAPDH	∞	3.14E-07
Hexokinase	HK3	∞	1.32E-03
L-serine dehydratase/L-threonine deaminase	SDS	∞	5.02E-05
Phosphoglycerate kinase 1	PGK1	∞	5.02E-05
Glycogen phosphorylase, liver form	PYGL	∞	9.62E-05
Pyruvate kinase	PKM	5.43	1.01E-11
Alpha-enolase	ENO1	2.84	1.06E-07
Chitinase-3-like protein 1	CHI3L1	1.89	5.29E-08
L-lactate dehydrogenase B chain	LDHB	1.85	6.27E-03
Alpha-lactalbumin	LALBA	− 1.40	7.44E-03
**B. Lipid metabolism**			
Annexin A1	ANXA1	∞	1.20E-09
Apolipoprotein A-IV	APOA4	∞	7.61E-06
Myeloperoxidase protein	MPO	∞	1.04E-06
Apolipoprotein E	APOE	3.17	7.93E-03
Perilipin	PLIN3	2.00	2.48E-03
Apolipoprotein A-I	APOA1	1.25	4.28E-05
Complement C3	C3	0.82	6.72E-04
Fatty acid synthase	FASN	− 0.60	4.13E-02
Platelet glycoprotein 4	CD36	− 0.69	8.70E-03
CIDE-N domain-containing protein	CIDEA	− 0.97	2.62E-02
Alpha-S1-casein	CSN1S1	− 1.09	5.82E-03
NPC intracellular cholesterol transporter 2	NPC2	− 1.15	1.39E-03
Lipoprotein lipase G	LIPG	− 1.47	3.65E-03
Lipoprotein lipase	LPL	− 2.00	2.98E-04
**C. Protein metabolism**			
Glyceraldehyde-3-phosphate dehydrogenase	GAPDH	∞	3.14E-07
Heat shock protein HSP 90-alpha	HSP90AA1	∞	1.04E-06
Leukocyte elastase inhibitor	SERPINB1	∞	5.03E-08
Myosin heavy chain 9	MYH9	∞	1.44E-05
Protein S100-A8	S100A8	∞	2.70E-05
SERPIN domain-containing protein	LOC786410	∞	9.03E-08
Pyruvate kinase	PKM	5.43	1.01E-11
Apolipoprotein E	APOE	3.17	7.93E-03
Antithrombin-III	SERPINC1	2.96	2.23E-07
Alpha-enolase	ENO1	2.84	1.06E-07
Alpha-1-antiproteinase	SERPINA1	2.46	6.78E-07
Fibronectin	FN1	2.25	1.18E-05
C4a anaphylatoxin	C4A	2.21	1.89E-16
Chitinase-3-like protein 1	CHI3L1	1.89	5.29E-08
Alpha-2-HS-glycoprotein	AHSG	1.74	9.08E-04
Gelsolin	GSN	1.44	1.51E-05
Moesin	MSN	1.40	1.59E-02
Serpin A3-6	SERPINA3-6	1.26	3.62E-03
Apolipoprotein A-I	APOA1	1.25	4.28E-05
Serpin A3-2	SERPINA3-2	1.08	1.89E-03
Serpin A3-7	SERPINA3-7	1.01	1.89E-04
Serpin A3-3	SERPINA3-3	0.92	2.81E-02
Complement C3	C3	0.82	6.72E-04
Clusterin	CLU	0.81	2.67E-02
Factor XIIa inhibitor	281,035	0.80	5.84E-03
Alpha-2-macroglobulin	A2M	0.76	7.44E-03
Serpin G1	SERPING1	0.73	8.30E-03
Lactotransferrin	LTF	0.63	1.30E-16
Inter-alpha-trypsin inhibitor heavy chain H4	ITIH4	0.59	2.56E-02
Metalloproteinase inhibitor 3	TIMP3	− 0.62	3.46E-02
Pigment epithelium-derived factor	SERPINF1	− 0.67	3.18E-02
Peptidyl-prolyl cis–trans isomerase A	PPIA	− 0.81	2.17E-03
Lipoprotein lipase G	LIPG	− 1.47	3.65E-03
**D. Immune system**			
Alpha-actinin-1	ACTN1	∞	5.02E-05
Annexin A1	ANXA1	∞	1.20E-09
Apolipoprotein A-IV	APOA4	∞	7.61E-06
Cathelicidin-4	CATHL4	∞	6.85E-04
Coronin-1A	CORO1A	∞	3.96E-06
Glucose-6-phosphate isomerase	GPI	∞	1.86E-04
Glyceraldehyde-3-phosphate dehydrogenase	GAPDH	∞	3.14E-07
Glycogen phosphorylase, liver form	PYGL	∞	9.62E-05
Haptoglobin	HP	∞	1.85E-11
Heat shock protein HSP 90-alpha	HSP90AA1	∞	1.04E-06
Ig-like domain-containing protein	ENSBTAG00000048030	∞	1.32E-03
Ig-like domain-containing protein	ENSBTAG00000050586	∞	2.70E-05
LRRCT domain-containing protein	LRG1	∞	9.62E-05
Myeloperoxidase	MPO	∞	1.04E-06
Myosin heavy chain 9	MYH9	∞	1.44E-05
Protein S100-A8	S100A8	∞	2.70E-05
Prothrombin	F2	∞	5.03E-08
Histone H2B type 1	VGNC:83,556	3.46	2.40E-03
Apolipoprotein E	APOE	3.17	7.93E-03
Peptidoglycan recognition protein 1	PGLYRP1	3.17	7.83E-06
Complement component C9	C9	2.93	3.65E-07
Cathelicidin-1	CATHL1	2.81	1.73E-06
Fibronectin	FN1	2.25	1.18E-05
C4a anaphylatoxin	C4A	2.21	1.89E-16
Chitinase-3-like protein 1	CHI3L1	1.89	5.29E-08
Alpha-2-HS-glycoprotein	AHSG	1.74	9.08E-04
Serotransferrin	TF	1.52	8.02E-28
Gelsolin	GSN	1.44	1.51E-05
Moesin	MSN	1.40	1.59E-02
Apolipoprotein A-I	APOA1	1.25	4.28E-05
Actin, cytoplasmic 2	ACTG1	1.14	5.04E-06
Lipocln_cytosolic_FA-bd_dom domain-containing protein	LCN2	1.08	1.63E-03
Ig-like domain-containing protein	ENSBTAG00000054702	1.04	7.44E-03
Ig-like domain-containing protein	ENSBTAG00000050373	0.92	2.86E-02
Complement C3	C3	0.82	6.72E-04
Clusterin	CLU	0.81	2.67E-02
Factor XIIa inhibitor	281,035	0.80	5.84E-03
Ig-like domain-containing protein	ENSBTAG00000050515	0.78	3.10E-02
SERPIN domain-containing protein	SERPING1	0.73	8.30E-03
Lactoperoxidase	LPO	0.66	2.58E-04
Fibrinogen beta chain	FGB	0.65	7.56E-03
Lactotransferrin	LTF	0.63	1.30E-16
Complement factor B	CFB	0.60	7.44E-03
Inter-alpha-trypsin inhibitor heavy chain H4	ITIH4	0.59	2.56E-02
Gamma-glutamyltransferase 1	GGT1	− 0.62	7.44E-03
Platelet glycoprotein 4	CD36	− 0.69	8.70E-03
Peptidyl-prolyl cis–trans isomerase A	PPIA	− 0.81	2.17E-03
Cytokeratin-1	KRT1	− 1.36	5.99E-03
Alpha-S2-casein	CSN1S2	− 5.06	3.63E-02

Groups of GO terms are related classes in an ontology with specific subclasses and parent terms are clustered; the list of all GO terms identified for H trial is available in Supplementary Data S3. The section on carbohydrate metabolism includes five different families of GO terms and 13 affected proteins. All these proteins except α-LA were more abundant in milk during feed restriction than during the pre-restriction period (Table 2A). The lipid metabolism section includes 29 different families of GO terms and 14 affected proteins (Table 2B). Their abundance in milk decreased for seven of these proteins and increased for the seven others during feed restriction. The protein metabolism section groups four different families of GO terms and 33 affected proteins (Table 2C). Most of these affected proteins were more abundant in milk during feed restriction than during the pre-restriction period. The immune system section groups 21 different families of GO terms and 49 affected proteins (Table 2D). Forty-four of these affected proteins were more abundant in milk during feed restriction than during the pre-restriction period, with 17 of them only being detected in milk during feed restriction.

## Discussion

The objective of this study was to investigate variations in milk protein composition induced by feed restriction, as well as the impact of the intensity of feed restriction on these variations. We first compared the milk protein composition in milk samples collected from the two trials before the feed restrictions were applied, in order to ensure that a comparison of both feed restriction trials was relevant. In terms of concentrations, the major milk protein profile was similar between the trials, although the concentration of α-LA was greater in M than H trial. This difference in α-LA concentration was most likely due to a difference in the lactation stage, as the cows involved in the M trial were around 77 DIM and those in H were around 165 DIM. Similarly, the yields of major protein were higher under M conditions than under H, particularly with respect to α-LA, αs1-, αs2- and β-CN, this being linked to higher milk yields in the M cows that were at peak lactation. Regarding proteomes, 345 proteins were identified during the trials, which was quite consistent with the milk proteomes published previously using LC–MS/MS. Indeed, among the 4654 proteins identified in the aggregation published by Delosière et al.^6^, 3288 were specific to colostrum and only 775 and 577 were identified during peak lactation and mid-lactation studies, respectively. After quantification and filtering, 23 low-abundance proteins were exclusive to the H trial and nine to the M trial during standard feeding. Again, this difference was very likely due to a difference in lactation stage as the proteome changes during lactation, with some proteins being exclusive to each stage^6^. In our trials, these 32 exclusive proteins only accounted for 0.9% of the total protein counts prior to restriction periods. The pre-restriction milk proteomes of both trials were therefore very similar and it was possible to compare their modifications induced by feed restriction. As the stage of lactation is not similar in both trials, the difference observed may be due to difference of physiological state of the cows, however all are in the declining phase of lactation. Since, in our comparison of feed restriction intensities, the lactation stage is a confound effect, we will thus focus on similarities rather than differences observed in the two trials.

In both trials, the reduction in milk yield induced by feed restriction was concomitant with a decrease in the major milk protein yield. This effect on major milk proteins increased in line with the intensity of the restriction. During the H trial, with an important negative energy balance (− 33.9 MJ/d) and milk yield loss (− 40%), all major milk proteins were quantitatively affected, whereas during the M trial, with lower negative energy balance (− 29.6 MJ/d) and milk yield loss (− 12%), only casein quantities were affected. During the high intensity feed restriction, this reduction in yield lowered the concentrations of αs1-, αs2- and β-CN. It appeared that the αs2-CN concentration was the most sensitive to feed restriction, as it was the most significantly affected during the H trial (− 25%) and tended to decrease under the M trial. When studying corn versus grass diets, Vanbergue et al.^3^ only observed variations in milk concentrations of αs2-CN (− 22%; *p* = 0.029) and β-CN (− 20%; *p* = 0.014), which supports the hypothesis that αs2-CN is the most sensitive to feed variation, followed by β- and αs1-CN. Billa et al.^16^ also saw a reduction in *CSN1S2* transcripts coding for αs2-CN in the mammary gland during the feed restriction period of the H trial. This shows that a reduction in the αs2-CN concentration in milk is directly linked to a decrease in *CSN1S2* gene expression in the mammary gland during a high intensity feed restriction.

Proteomic analyses confirmed the decreased concentrations of α-LA, αs1- and αs2-CN during H feed restriction, with αs2-CN being the most affected protein. This analysis also showed a significant effect of intense feed restriction on proteins involved in lipid metabolism, with 14 affected proteins involved in this metabolism in the H trial. Among the seven proteins involved in this metabolism which displayed increased abundance during feed restriction, four involved in lipid transport and storage were found: apolipoproteins (A-I, A-IV and E) and perilipin. Moreover, the decreased abundance of CIDE-N domain-containing protein, a lipolysis inhibitor and storage activator, may have reflected increased lipid mobilization in adipose tissue, which is consistent with the increase in plasma NEFA concentrations observed in both trials. Lower concentrations of fatty acid synthase, which catalyzes the de novo biosynthesis of fatty acids, were observed in milk under H conditions. This finding was in line with the reported decrease of FASN RNA in the cytosolic crescent of milk fat globules during 40% feed restriction over four days^17^. It was also consistent with the decrease in de novo synthesized fatty acids during the H trial, reflected by the reduction in short chain fatty acid concentrations in milk^7^. A rise in the fat content of up to 13% was due to the uptake of long chain fatty acids from lipid mobilization, as indicated by the increase in plasma NEFA concentrations seen during both trials. Such adaptations of lipid metabolism in the context of a negative energy balance have already been well described^1,18,19^, and notably involved the downregulation of several mammary lipogenic genes during the first days of short-term feed restriction^17^. However, during our M trial, concentrations of fatty acid synthase rose slightly after five days of feed restriction, suggesting that intense restriction is necessary for this shift in fatty acid metabolism to occur.

The modifications observed regarding milk proteins and proteome were in response to changes to mammary metabolism, partly because of a reduction in nutrient uptake by the mammary gland during feed restriction, as shown previously by Guinard-Flament et al.^20^. Indeed, these authors showed that feed restriction reduced mammary blood flow alongside reductions in mammary nutrient and dioxygen uptakes during a − 30% DMI feed restriction^20^. Nevertheless, under H conditions in our study, mammary metabolism appeared to partially compensate for decreased nutrient uptake by increasing carbohydrate catabolism and lipid transport. Indeed, among the 12 proteins involved in carbohydrate metabolism that were more abundant in milk during the H trial, seven are involved in glycolysis (hexokinase, glucose-6-phosphate isomerase, fructose-bisphosphate aldolase, glyceraldehyde-3-phosphate dehydrogenase, phosphoglycerate kinase 1, alpha-enolase and pyruvate kinase), one is involved in the pentose phosphate pathway (6-phosphogluconate dehydrogenase, decarboxylating), which is the parallel pathway to glycolysis, and glycogen phosphorylase catalyzes the rate-limiting step in glycogenolysis that produces substrate for both the glycolysis and pentose phosphate pathways. This increased abundance of proteins involved in carbohydrate degradation in milk may reflect the high level of energy required to maintain mammary gland metabolism in a lactating cow.

The total protein content decreased only during H trial by 4%, despite the lower concentrations of some major milk proteins. Lacy-Hulbert et al.^21^, who had observed an increased total protein content in milk (+ 8%) during intense feed restriction (− 50% of DMI for 26 days) hypothesized that feed restriction tended to concentrate serum-derived proteins in milk. Indeed, 43 proteins identified in the H milk samples during feed restriction had not been present before restriction, and among the seven proteins with increased concentrations in milk under both the M and H conditions, five are normally present in plasma (ceruloplasmin, apolipoprotein A-IV, alpha-1B-glycoprotein, angiotensinogen and serotransferrin). This increase in plasma protein concentrations in milk may reflect a loss of mammary epithelial barrier integrity, which could play a role in reducing milk production during feed restriction. This had already been suggested by Herve et al.^8^ who observed an elevated rate of mammary epithelial cell exfoliation under M trial, as well as an increased Na^+^ concentration in milk, and by Stumpf et al.^22^ who saw an increased permeability of mammary cell tight junctions during short and intense feed restriction (− 50% DMI for seven days). Under our H conditions, we observed an elevation of lactotransferrin concentrations in milk, an increase that is known to happen during the first days of the dry period^23^ when involution starts and the epithelial barrier loses its integrity. This increased permeability of the epithelial barrier is coupled with increased leucocyte infiltration of the mammary gland, as shown by higher milk somatic cell count in the M trial^8^ and during other feed restriction experiments^21,24–26^, and an upregulation of immune genes, as observed in the mammary tissue during involution^27^. Moreover, 12 of the 13 proteins involved in positive regulation of immune system processes were more abundant in milk during the feed restriction period under H conditions, suggesting a similar immune system upregulation. Variations in milk of the immune system related protein were confirmed for five of them, with similar changes to their transcript levels in the mammary gland^16^, and in particular C3, which plays a central role in activation of the complement system. However, among the 33 proteins involved in protein metabolism, 12 over-abundant proteins have a protease inhibition function (α-1-antiproteinase, α-2-macroglobulin, antithrombin-III, factor XIIa inhibitor, inter-α-inhibitor heavy chain H4, leukocyte elastase inhibitor and serpins A3-2, A3-3, A3-6, A3-7, B4 and G1) and four are involved in the inhibition of complement activation, inflammation and cell death (chitinase-3-like protein 1, clusterin, factor XIIa inhibitor and heat shock protein HSP 90-α). These results therefore suggest a greater regulation of the immune system in the mammary gland during feed restriction.

These adaptations, which are reminiscent of some of those observed during early involution, remained reversible during these restriction trials, as both milk yield and composition recovered after a return to ad libitum feeding. Delosière et al.^6^ proposed some milk proteins exclusive to early lactation as biomarkers of negative energy balance, and none of these were found in milk during the negative energy balance induced by feed restriction later in lactation. Nevertheless, some proteins are affected by both moderate and high intensity feed restrictions: alpha-enolase, ceruloplasmin, apolipoprotein A-IV, alpha-1B-glycoprotein, angiotensinogen and serotransferrin.

Alpha-enolase is an enzyme present in all tissues that catalyzes the interconversion of 2-phosphoglycerate to phosphoenolpyruvate; its upregulation indicates an enhancement of glycolysis and has also been observed during ketosis^28^, a common metabolic disease induced by a negative energy balance. The five other proteins affected by both moderate and intense feed restriction were mainly found secreted in plasma. Apolipoprotein A-IV is primarily synthesized in the small intestine; this lipid-binding protein is involved in numerous physiological processes such as lipid metabolism and glucose homeostasis^29^. Apolipoprotein A-IV upregulation in the bovine mammary gland has been described during inflammation challenges where its anti-inflammatory activities may balance the immune response^30^. Ceruloplasmin, alpha-1B-glycoprotein, serotransferrin and angiotensinogen are mainly expressed in the liver. Ceruloplasmin is a copper-binding glycoprotein with antioxidant and cytoprotective activities. Increased concentrations of cerulaplasmin in bovine milk have been described during subclinical and clinical mastitis^31^ and may indicate inflammation. Alpha-1B-glycoprotein is a glycoprotein of unknown function. In the cow, its serum level seems to increase during various stresses such as tuberculosis^32^, high-altitude hypoxia^33^ or mastitis^34^. Serotransferrin, an iron binding transport glycoprotein, is seen at high concentrations in milk during early lactation, and then fall rapidly over time. Mastitis events can also increase serotransferrin concentrations in milk through changes to the mammary gland epithelium^35^. Angiotensinogen is the precursor of angiotensin. In dairy cows it has been shown that ketosis may alter the metabolism of angiotensinogen to angiotensin^36^.

## Conclusion

Feed restriction induced modifications to the milk protein composition even if there was little or no decrease in the total milk protein content. Feed restriction reduced the yield of major milk proteins, only affecting caseins when the restriction was of moderate intensity. Low-abundance protein concentrations were also affected by feed restriction. Six of them were similarly affected by feed restrictions of high and moderate intensities, regardless of the lactation stage, of which five are normally present in plasma. These proteome variations reflected mammary gland adaptation to this stress with a loss of mammary epithelial barrier integrity and altered immune function, sharing common features with the changes observed during the early phase of mammary gland involution. The six low-abundance proteins that were affected by both moderate and high intensity feed restrictions, as well as αs2-CN, are putative biomarkers of a negative energy balance in dairy cows.

## Supplementary Information

Supplementary Information 1.

Supplementary Information 2.

## Data Availability

The proteomic data are available on the ProteomeXchange Consortium via the PRIDE partner repository with the dataset identifier PXD033969. Gene Ontology datasets analyzed during the current study are included in the Supplementary Data of this published article, HPLC raw datasets are available from the corresponding author on reasonable request.
