# Supplementary material for: Milk proteins as a feed restriction signature indicating the metabolic adaptation of dairy cows
Source: Sci Rep. 2022 Nov 7;12:18886. doi: 10.1038/s41598-022-21804-1 (PMC9640695; doi:10.1038/s41598-022-21804-1)
Supplement: Supplementary file 1 — Supplementary Information 1. [file 41598_2022_21804_MOESM1_ESM.docx]

Supplementary Data Leduc et al. Submitted to Scientific report

Supplementary Data S1: Quantification of skim milk proteins identified by LC-MS/MS before (ctrl) and after five days (res) of two feed restriction trials: one of moderate intensity (M: 8 Holstein cows, -18% of dry matter intake) and the other of high intensity (H: 8 Holstein cows, ‑66% of dry matter intake). A: the 345 identified proteins and their spectral count for each sample. B: the 232 remaining proteins after filtration (peptides with E-value <0.01, proteins with log (E-value) <−4 and a minimum of two identified peptides per protein) and their spectral count for each sample.

Supplementary Data S2: List of all 160 skim milk proteins whose abundance varied (*P* <0.05) during the feed restriction trial of high intensity (-66% dry matter intake) with their respective fold change and adjusted *P*-value. Fold change (FC) is the log2 ratio between d 5 and d -2 relative to feed restriction initiation. ∞ represent proteins only found in milk during feed restriction and -∞ those only found before feed restriction.

| Protein name | Accession | log2 FC | Adj p.value |
| --- | --- | --- | --- |
| Transcobalamin-2 | TCO2_BOVIN | -∞ | 4E-04 |
| Alpha-S2-casein | CASA2_BOVIN | -5.03 | 0.04 |
| Lipoprotein lipase | LIPL_BOVIN | -1.98 | 3E-04 |
| Dehydrogenase/reductase (SDR family) member 1 | Q2KIS4_BOVIN | -1.58 | 4E-03 |
| Uncharacterized protein | A0A3Q1LR40_BOVIN | -1.49 | 4E-03 |
| Alpha-lactalbumin | LALBA_BOVIN | -1.41 | 0.01 |
| Keratin 1 | G3N0V2_BOVIN | -1.35 | 0.01 |
| Alpha-S1-casein | CASA1_BOVIN | -1.34 | 0.01 |
| Keratin, type I cytoskeletal 10 | K1C10_BOVIN | -1.30 | 0.01 |
| Glycosylation-dependent cell adhesion molecule 1 | GLCM1_BOVIN | -1.24 | 0.01 |
| NPC intracellular cholesterol transporter 2 | NPC2_BOVIN | -1.16 | 1E-03 |
| Serine protease HTRA1 | HTRA1_BOVIN | -1.14 | 0.01 |
| Synaptobrevin homolog YKT6 | YKT6_BOVIN | -1.11 | 0.01 |
| CIDEA protein | A4FUX1_BOVIN | -0.98 | 0.03 |
| Ras-related protein Rab-11A | RB11A_BOVIN | -0.98 | 1E-03 |
| Sodium-dependent phosphate transport protein 2B | A0A3Q1MG98_BOVIN | -0.95 | 0.01 |
| GTP-binding protein SAR1a | SAR1A_BOVIN | -0.93 | 0.01 |
| ADP-ribosylation factor 1 | ARF1_BOVIN | -0.90 | 3E-03 |
| 1-acyl-sn-glycerol-3-phosphate acyltransferase alpha | PLCA_BOVIN | -0.88 | 0.01 |
| Nucleoside diphosphate kinase B | NDKB_BOVIN | -0.86 | 0.01 |
| Peptidyl-prolyl cis-trans isomerase A | PPIA_BOVIN | -0.80 | 2E-03 |
| Lactadherin | F1MXX6_BOVIN | -0.78 | 0.02 |
| Mucin-1 | MUC1_BOVIN | -0.78 | 0.01 |
| C11H2ORF7 protein | A6QQW5_BOVIN | -0.71 | 0.01 |
| Sulfhydryl oxidase | A0A3Q1N9Y5_BOVIN | -0.71 | 0.01 |
| ATP-binding cassette sub-family G member 2 | ABCG2_BOVIN | -0.70 | 0.04 |
| Platelet glycoprotein 4 | CD36_BOVIN | -0.70 | 0.01 |
| Fibroblast growth factor-binding protein 1 | FGFP1_BOVIN | -0.70 | 0.01 |
| Ras-related protein Rab-7a | A0A3Q1M3K7_BOVIN | -0.69 | 0.01 |
| NADH-cytochrome b5 reductase 3 | NB5R3_BOVIN | -0.67 | 0.03 |
| Pigment epithelium-derived factor | PEDF_BOVIN | -0.67 | 0.03 |
| Glycoprotein 2 | A0A3Q1M193_BOVIN | -0.66 | 0.03 |
| Ras-related protein Rap-1b | RAP1B_BOVIN | -0.65 | 0.01 |
| RAB1A, member RAS oncogene family | A0A3Q1LYE7_BOVIN | -0.64 | 0.01 |
| 5'-nucleotidase | 5NTD_BOVIN | -0.64 | 0.03 |
| Metalloproteinase inhibitor 3 | TIMP3_BOVIN | -0.63 | 0.03 |
| Uncharacterized protein | G3N2D8_BOVIN | -0.62 | 0.01 |
| Rab GDP dissociation inhibitor beta | GDIB_BOVIN | -0.60 | 0.03 |
| Fatty acid synthase | F1N647_BOVIN | -0.60 | 0.04 |
| Heat shock cognate 71 kDa protein | HSP7C_BOVIN | -0.60 | 3E-03 |
| Inter-alpha-trypsin inhibitor heavy chain H4 | ITIH4_BOVIN | 0.60 | 0.03 |
| Complement factor B | CFAB_BOVIN | 0.60 | 0.01 |
| Lactotransferrin | TRFL_BOVIN | 0.63 | 1E-16 |
| Lactoferrin | B3VTM3_BOVIN | 0.63 | 1E-16 |
| Fibrinogen beta chain | A0A3Q1MG04_BOVIN | 0.65 | 0.01 |
| Lactoperoxidase | PERL_BOVIN | 0.66 | 3E-04 |
| Lipopolysaccharide-binding protein | LBP_BOVIN | 0.68 | 0.01 |
| Immunoglobulin light chain, lambda gene cluster | Q1RMN8_BOVIN | 0.70 | 2E-04 |
| Serpin family G member 1 | E1BMJ0_BOVIN | 0.73 | 0.01 |
| Uncharacterized protein | A0A3Q1M3L6_BOVIN | 0.75 | 2E-05 |
| Alpha-2-macroglobulin | A2MG_BOVIN | 0.76 | 0.01 |
| Uncharacterized protein | A5PK72_BOVIN | 0.77 | 4E-04 |
| Uncharacterized protein | A0A3Q1LUE9_BOVIN | 0.78 | 0.03 |
| IGL@ protein | Q3T101_BOVIN | 0.79 | 0.04 |
| Uncharacterized protein | F1MLW8_BOVIN | 0.79 | 0.02 |
| Alpha-1B-glycoprotein | A1BG_BOVIN | 0.80 | 7E-04 |
| Elongation factor 1-alpha 1 | EF1A1_BOVIN | 0.80 | 0.03 |
| Factor XIIa inhibitor | F12AI_BOVIN | 0.80 | 0.01 |
| Uncharacterized protein | A5D7Q2_BOVIN | 0.81 | 5E-03 |
| Clusterin | CLUS_BOVIN | 0.81 | 0.03 |
| Complement C3 | CO3_BOVIN | 0.82 | 7E-04 |
| Angiotensinogen | A0A3Q1LGY9_BOVIN | 0.88 | 0.01 |
| Uncharacterized protein | A0A3Q1LSF0_BOVIN | 0.92 | 0.03 |
| Serpin A3-3 | G3N1U4_BOVIN | 0.92 | 0.03 |
| Uncharacterized protein | A0A3Q1LPG0_BOVIN | 0.97 | 4E-03 |
| Vitamin D-binding protein | VTDB_BOVIN | 1.00 | 0.01 |
| IGK protein | B0JYP6_BOVIN | 1.00 | 2E-03 |
| Uncharacterized protein | G3N0V0_BOVIN | 1.01 | 4E-03 |
| Uncharacterized protein | A0A3Q1ML26_BOVIN | 1.04 | 0.01 |
| DnaJ homolog subfamily C member 3 | DNJC3_BOVIN | 1.04 | 0.02 |
| Uncharacterized protein | G5E5T5_BOVIN | 1.05 | 0.03 |
| Uncharacterized protein | F1MZ96_BOVIN | 1.05 | 2E-03 |
| Lipocalin 2 | E1B6Z6_BOVIN | 1.08 | 2E-03 |
| Serpin A3-2 | SPA32_BOVIN | 1.08 | 2E-03 |
| Uncharacterized protein | A0A3Q1M032_BOVIN | 1.10 | 0.02 |
| Endopin 2C | Q32T06_BOVIN | 1.13 | 0.01 |
| Actin, cytoplasmic 2 | ACTG_BOVIN | 1.14 | 5E-06 |
| Uncharacterized protein | A0A3Q1NEQ0_BOVIN | 1.14 | 0.01 |
| Actin, cytoplasmic 1 | ACTB_BOVIN | 1.15 | 4E-06 |
| Uncharacterized protein | A0A3Q1NKP5_BOVIN | 1.17 | 0.01 |
| Serpin A3-7 | G8JKW7_BOVIN | 1.20 | 1E-04 |
| Apolipoprotein A-I | APOA1_BOVIN | 1.25 | 4E-05 |
| Serpin A3-6 | SPA36_BOVIN | 1.26 | 4E-03 |
| Thrombospondin-1 | A0A3Q1MQV3_BOVIN | 1.26 | 2E-04 |
| Pancreatic adenocarcinoma upregulated factor-like | F1N1Z8_BOVIN | 1.30 | 2E-03 |
| Fibrinogen alpha chain | FIBA_BOVIN | 1.31 | 5E-06 |
| Actin, alpha skeletal muscle | ACTS_BOVIN | 1.38 | 8E-05 |
| Moesin | MOES_BOVIN | 1.39 | 0.02 |
| Gelsolin | GELS_BOVIN | 1.44 | 2E-05 |
| Serotransferrin | TRFE_BOVIN | 1.54 | 2E-28 |
| Isoform LMW of Kininogen-2 | KNG2_BOVIN | 1.64 | 2E-03 |
| Acyl-CoA synthetase long chain family member 1 | A0A3Q1LVU8_BOVIN | 1.70 | 2E-03 |
| Alpha-2-HS-glycoprotein | FETUA_BOVIN | 1.74 | 9E-04 |
| Histone H4 | H4_BOVIN | 1.75 | 1E-03 |
| Kininogen-1 | F1MNV5_BOVIN | 1.81 | 1E-03 |
| L-lactate dehydrogenase B chain | LDHB_BOVIN | 1.85 | 0.01 |
| Chitinase-3-like protein 1 | CH3L1_BOVIN | 1.89 | 5E-08 |
| Cofilin-1 | COF1_BOVIN | 1.94 | 2E-03 |
| Perilipin | Q3SX32_BOVIN | 2.00 | 2E-03 |
| Tubulin alpha-1B chain | TBA1B_BOVIN | 2.12 | 0.01 |
| Uncharacterized protein | F1MVK1_BOVIN | 2.15 | 1E-16 |
| Uncharacterized protein | E1BH06_BOVIN | 2.21 | 2E-16 |
| Fibronectin | FINC_BOVIN | 2.25 | 1E-05 |
| Alpha-1-antiproteinase | A1AT_BOVIN | 2.46 | 7E-07 |
| Rab GDP dissociation inhibitor alpha | GDIA_BOVIN | 2.66 | 6E-04 |
| Cathelicidin-1 | CTHL1_BOVIN | 2.81 | 2E-06 |
| Alpha-enolase | ENOA_BOVIN | 2.84 | 1E-07 |
| Complement component C9 | CO9_BOVIN | 2.93 | 4E-07 |
| Antithrombin-III | A0A3Q1NJR8_BOVIN | 2.96 | 2E-07 |
| Alpha-2-macroglobulin variant 22 | K4JR88_BOVIN | 3.00 | 9E-09 |
| Uncharacterized protein | G3MZ19_BOVIN | 3.06 | 2E-05 |
| Apolipoprotein E | APOE_BOVIN | 3.17 | 0.01 |
| Peptidoglycan recognition protein 1 | PGRP1_BOVIN | 3.17 | 8E-06 |
| Histone H2B type 1 | H2B1_BOVIN | 3.46 | 2E-03 |
| Histone H2B | A0A3Q1MBN5_BOVIN | 3.46 | 2E-03 |
| Hemopexin | HEMO_BOVIN | 4.09 | 7E-05 |
| Pyruvate kinase | A5D984_BOVIN | 5.43 | 1E-11 |
| Lymphocyte cytosolic protein 1 | A0A3Q1LSN0_BOVIN | ∞ | 6E-12 |
| Haptoglobin | HPT_BOVIN | ∞ | 2E-11 |
| Annexin A1 | ANXA1_BOVIN | ∞ | 1E-09 |
| Uncharacterized protein | A0A3Q1LMW6_BOVIN | ∞ | 2E-09 |
| Alpha-2-macroglobulin variant 5 | K4JDR8_BOVIN | ∞ | 8E-09 |
| Uncharacterized protein | F1MMS7_BOVIN | ∞ | 3E-08 |
| Prothrombin | THRB_BOVIN | ∞ | 5E-08 |
| Leukocyte elastase inhibitor | ILEU_BOVIN | ∞ | 5E-08 |
| Uncharacterized protein | G3X8C3_BOVIN | ∞ | 9E-08 |
| Glyceraldehyde-3-phosphate dehydrogenase | G3P_BOVIN | ∞ | 3E-07 |
| Alpha-actinin-4 | ACTN4_BOVIN | ∞ | 1E-06 |
| Heat shock protein HSP 90-alpha | HS90A_BOVIN | ∞ | 1E-06 |
| ELA2 protein | A6QPP7_BOVIN | ∞ | 1E-06 |
| MPO protein | A6QPT4_BOVIN | ∞ | 1E-06 |
| Coronin-1A | COR1A_BOVIN | ∞ | 4E-06 |
| Apolipoprotein A-IV | APOA4_BOVIN | ∞ | 8E-06 |
| Fructose-bisphosphate aldolase | A0A3Q1LMG1_BOVIN | ∞ | 1E-05 |
| Myosin heavy chain 9 | F1MQ37_BOVIN | ∞ | 1E-05 |
| Protein S100-A8 | S10A8_BOVIN | ∞ | 3E-05 |
| Uncharacterized protein | A0A3Q1MIN7_BOVIN | ∞ | 3E-05 |
| L-serine dehydratase/L-threonine deaminase | SDHL_BOVIN | ∞ | 5E-05 |
| Alpha-actinin-1 | ACTN1_BOVIN | ∞ | 5E-05 |
| Phosphoglycerate kinase 1 | PGK1_BOVIN | ∞ | 5E-05 |
| Azurocidin 1 | G3N0Q8_BOVIN | ∞ | 5E-05 |
| Glycogen phosphorylase, liver form | PYGL_BOVIN | ∞ | 1E-04 |
| 6-phosphogluconate dehydrogenase, decarboxylating | A0A3S5ZPM3_BOVIN | ∞ | 1E-04 |
| Leucine rich alpha-2-glycoprotein 1 | F6RMV5_BOVIN | ∞ | 1E-04 |
| Adenylyl cyclase-associated protein 1 | CAP1_BOVIN | ∞ | 2E-04 |
| Glucose-6-phosphate isomerase | G6PI_BOVIN | ∞ | 2E-04 |
| Hemoglobin subunit beta | HBB_BOVIN | ∞ | 4E-04 |
| Protein S100-A9 | S10A9_BOVIN | ∞ | 4E-04 |
| Cathelicidin-4 | CTHL4_BOVIN | ∞ | 7E-04 |
| TKT protein | A5PJ79_BOVIN | ∞ | 7E-04 |
| WD repeat-containing protein 1 | WDR1_BOVIN | ∞ | 1E-03 |
| Hexokinase 3 | A0A3Q1LR63_BOVIN | ∞ | 1E-03 |
| Peptidyl arginine deiminase 4 | E1BCN3_BOVIN | ∞ | 1E-03 |
| Aminopeptidase | E1BP91_BOVIN | ∞ | 1E-03 |
| Uncharacterized protein | G3MZE0_BOVIN | ∞ | 1E-03 |
| Ceruloplasmin | A0A3Q1NJB1_BOVIN | ∞ | 1E-03 |
| SERPIND1 protein | A6QPP2_BOVIN | ∞ | 1E-03 |
| Hemoglobin subunit alpha | HBA_BOVIN | ∞ | 2E-03 |
| Histone H1.2 | H12_BOVIN | ∞ | 2E-03 |
| Threonine-tRNA ligase, cytoplasmic | SYTC_BOVIN | ∞ | 5E-03 |

Supplementary Data S3: List of all Gene Ontology terms identified from the 160 skim milk proteins presenting abundance variation after five days of a feed restriction trial of high intensity (-66% dry matter intake) with their respective *P*-value and false discovery rate (FDR) as well as the metabolic pathway group in which they were included. Results are sorted hierarchically with the most specific subclass first and its parent terms indented directly below it.

| GO biological process complete | #proteins | *P*-value | FDR | Metabolic pathway group |
| --- | --- | --- | --- | --- |
| uropod organization | 2 | 1.76E-04 | 1.23E-02 |  |
| cellular component organization | 43 | 6.39E-04 | 3.58E-02 |  |
| positive regulation of protein processing in phagocytic vesicle | 2 | 1.76E-04 | 1.23E-02 | immune system |
| regulation of protein processing in phagocytic vesicle | 2 | 1.76E-04 | 1.22E-02 | immune system |
| regulation of protein metabolic process | 36 | 6.50E-10 | 2.92E-07 | protein metabolism |
| regulation of primary metabolic process | 46 | 2.28E-04 | 1.51E-02 |  |
| regulation of metabolic process | 56 | 4.60E-06 | 5.90E-04 |  |
| biological regulation | 87 | 2.04E-04 | 1.38E-02 |  |
| regulation of macromolecule metabolic process | 53 | 7.04E-06 | 8.50E-04 |  |
| regulation of proteolysis | 29 | 7.57E-18 | 1.36E-14 | protein metabolism |
| positive regulation of protein processing | 3 | 3.75E-04 | 2.27E-02 |  |
| positive regulation of cellular process | 42 | 7.62E-04 | 4.13E-02 |  |
| positive regulation of biological process | 54 | 2.44E-07 | 5.32E-05 |  |
| positive regulation of metabolic process | 31 | 9.17E-04 | 4.71E-02 |  |
| regulation of cellular metabolic process | 49 | 6.70E-05 | 5.80E-03 |  |
| regulation of cellular protein metabolic process | 35 | 4.15E-10 | 2.06E-07 | protein metabolism |
| positive regulation of protein maturation | 3 | 4.69E-04 | 2.76E-02 | protein metabolism |
| positive regulation of gene expression | 19 | 5.28E-07 | 1.02E-04 |  |
| positive regulation of phospholipid efflux | 2 | 2.92E-04 | 1.87E-02 | lipid metabolism |
| regulation of lipid transport | 5 | 1.58E-04 | 1.17E-02 | lipid metabolism |
| regulation of transport | 22 | 1.34E-06 | 2.14E-04 |  |
| regulation of localization | 29 | 3.57E-06 | 4.75E-04 |  |
| regulation of lipid localization | 9 | 2.49E-08 | 6.74E-06 | lipid metabolism |
| positive regulation of lipid transport | 4 | 3.04E-04 | 1.90E-02 | lipid metabolism |
| positive regulation of transport | 13 | 3.33E-05 | 3.28E-03 |  |
| positive regulation of lipid localization | 8 | 1.59E-08 | 4.47E-06 | lipid metabolism |
| regulation of phospholipid efflux | 2 | 2.92E-04 | 1.86E-02 | lipid metabolism |
| response to 11-deoxycorticosterone | 3 | 8.69E-06 | 1.01E-03 |  |
| response to mineralocorticoid | 3 | 3.36E-05 | 3.28E-03 |  |
| response to corticosteroid | 4 | 1.73E-04 | 1.25E-02 |  |
| response to organic substance | 24 | 3.40E-04 | 2.11E-02 |  |
| response to chemical | 38 | 1.72E-04 | 1.25E-02 |  |
| response to stimulus | 75 | 5.73E-10 | 2.66E-07 |  |
| response to lipid | 9 | 5.95E-04 | 3.39E-02 | lipid metabolism |
| response to ketone | 4 | 9.68E-04 | 4.93E-02 |  |
| response to oxygen-containing compound | 14 | 8.64E-04 | 4.50E-02 |  |
| response to dehydroepiandrosterone | 3 | 8.69E-06 | 9.99E-04 |  |
| very-low-density lipoprotein particle remodeling | 4 | 2.70E-07 | 5.79E-05 | lipid metabolism |
| triglyceride-rich lipoprotein particle remodeling | 4 | 4.03E-07 | 8.28E-05 | lipid metabolism |
| plasma lipoprotein particle remodeling | 6 | 6.28E-09 | 2.05E-06 | lipid metabolism |
| plasma lipoprotein particle organization | 6 | 3.97E-08 | 1.02E-05 | lipid metabolism |
| protein-lipid complex subunit organization | 6 | 6.56E-08 | 1.57E-05 | lipid metabolism |
| protein-containing complex subunit organization | 20 | 2.10E-05 | 2.17E-03 |  |
| protein-lipid complex remodeling | 6 | 6.28E-09 | 2.01E-06 | lipid metabolism |
| protein-containing complex remodeling | 6 | 9.99E-09 | 2.99E-06 |  |
| regulation of plasma lipoprotein particle levels | 7 | 8.60E-09 | 2.63E-06 | lipid metabolism |
| regulation of transepithelial transport | 2 | 4.36E-04 | 2.60E-02 |  |
| postsynaptic actin cytoskeleton organization | 3 | 1.85E-05 | 1.97E-03 |  |
| actin cytoskeleton organization | 12 | 2.29E-05 | 2.35E-03 |  |
| actin filament-based process | 12 | 4.70E-05 | 4.30E-03 |  |
| postsynaptic cytoskeleton organization | 3 | 2.53E-05 | 2.58E-03 |  |
| postsynapse organization | 6 | 1.35E-06 | 2.13E-04 |  |
| synapse organization | 7 | 1.71E-04 | 1.25E-02 |  |
| high-density lipoprotein particle assembly | 3 | 1.85E-05 | 1.95E-03 | lipid metabolism |
| plasma lipoprotein particle assembly | 3 | 1.44E-04 | 1.11E-02 | lipid metabolism |
| protein-lipid complex assembly | 3 | 2.26E-04 | 1.51E-02 | lipid metabolism |
| peptidyl-cysteine S-nitrosylation | 2 | 6.08E-04 | 3.46E-02 |  |
| protein nitrosylation | 2 | 8.08E-04 | 4.32E-02 |  |
| reverse cholesterol transport | 4 | 8.09E-07 | 1.45E-04 | lipid metabolism |
| cholesterol transport | 6 | 4.86E-07 | 9.56E-05 | lipid metabolism |
| sterol transport | 6 | 2.50E-06 | 3.52E-04 | lipid metabolism |
| lipid localization | 9 | 6.83E-05 | 5.84E-03 | lipid metabolism |
| organic hydroxy compound transport | 6 | 9.08E-05 | 7.46E-03 |  |
| positive regulation of cholesterol esterification | 3 | 2.53E-05 | 2.56E-03 | lipid metabolism |
| regulation of cholesterol esterification | 3 | 3.36E-05 | 3.24E-03 | lipid metabolism |
| regulation of steroid metabolic process | 4 | 8.39E-04 | 4.40E-02 |  |
| regulation of lipid metabolic process | 7 | 6.97E-04 | 3.87E-02 | lipid metabolism |
| positive regulation of steroid metabolic process | 4 | 8.23E-06 | 9.69E-04 |  |
| positive regulation of lipid metabolic process | 5 | 4.95E-04 | 2.89E-02 | lipid metabolism |
| regulation of Cdc42 protein signal transduction | 2 | 8.08E-04 | 4.35E-02 |  |
| regulation of response to stimulus | 34 | 5.71E-05 | 5.09E-03 |  |
| cholesterol import | 2 | 8.08E-04 | 4.33E-02 | lipid metabolism |
| sterol import | 2 | 8.08E-04 | 4.30E-02 | lipid metabolism |
| positive regulation of sequestering of triglyceride | 3 | 3.36E-05 | 3.26E-03 | lipid metabolism |
| positive regulation of lipid storage | 4 | 9.75E-06 | 1.10E-03 | lipid metabolism |
| regulation of lipid storage | 4 | 8.82E-05 | 7.33E-03 | lipid metabolism |
| regulation of sequestering of triglyceride | 3 | 1.02E-04 | 8.22E-03 | lipid metabolism |
| complement activation, alternative pathway | 3 | 5.51E-05 | 4.98E-03 | immune system |
| innate immune response | 20 | 1.08E-11 | 6.75E-09 | immune system |
| defense response to other organism | 28 | 1.50E-15 | 1.27E-12 | immune system |
| response to other organism | 35 | 1.93E-18 | 5.55E-15 | immune system |
| biological process involved in interspecies interaction between organisms | 38 | 6.48E-20 | 9.32E-16 | immune system |
| response to external biotic stimulus | 35 | 2.11E-18 | 5.06E-15 | immune system |
| response to biotic stimulus | 35 | 5.08E-18 | 1.04E-14 | immune system |
| response to external stimulus | 39 | 2.66E-14 | 2.12E-11 | immune system |
| defense response | 33 | 7.66E-17 | 1.00E-13 | immune system |
| response to stress | 44 | 1.43E-12 | 9.80E-10 | immune system |
| immune response | 35 | 3.55E-16 | 3.64E-13 | immune system |
| immune system process | 42 | 4.19E-16 | 4.01E-13 | immune system |
| complement activation | 8 | 3.11E-09 | 1.17E-06 | immune system |
| immune effector process | 12 | 1.99E-07 | 4.39E-05 | immune system |
| activation of immune response | 9 | 2.16E-06 | 3.17E-04 | immune system |
| positive regulation of immune response | 10 | 7.43E-05 | 6.21E-03 | immune system |
| positive regulation of response to stimulus | 26 | 9.79E-07 | 1.72E-04 | immune system |
| regulation of immune response | 15 | 5.12E-06 | 6.34E-04 | immune system |
| regulation of immune system process | 21 | 1.15E-06 | 1.88E-04 | immune system |
| positive regulation of immune system process | 13 | 1.05E-04 | 8.43E-03 | immune system |
| humoral immune response | 18 | 1.95E-16 | 2.16E-13 | immune system |
| phospholipid efflux | 3 | 6.87E-05 | 5.84E-03 | lipid metabolism |
| high-density lipoprotein particle remodeling | 3 | 6.87E-05 | 5.80E-03 | lipid metabolism |
| acute-phase response | 5 | 5.68E-07 | 1.09E-04 |  |
| acute inflammatory response | 5 | 5.68E-06 | 6.92E-04 | immune system |
| inflammatory response | 11 | 2.13E-06 | 3.15E-04 | immune system |
| response to progesterone | 3 | 1.22E-04 | 9.50E-03 |  |
| glycolytic process | 7 | 3.31E-09 | 1.16E-06 | carbohydrate metabolism |
| carbohydrate catabolic process | 9 | 1.89E-09 | 7.77E-07 | carbohydrate metabolism |
| carbohydrate metabolic process | 13 | 6.44E-07 | 1.22E-04 | carbohydrate metabolism |
| organic substance catabolic process | 20 | 1.52E-04 | 1.15E-02 |  |
| catabolic process | 26 | 2.17E-06 | 3.15E-04 |  |
| ATP generation from ADP | 7 | 3.31E-09 | 1.19E-06 |  |
| ADP metabolic process | 7 | 1.15E-08 | 3.37E-06 |  |
| purine ribonucleotide metabolic process | 8 | 1.50E-04 | 1.13E-02 |  |
| purine nucleotide metabolic process | 8 | 2.00E-04 | 1.37E-02 |  |
| nucleotide metabolic process | 9 | 2.43E-04 | 1.59E-02 |  |
| nucleoside phosphate metabolic process | 9 | 3.05E-04 | 1.90E-02 |  |
| small molecule metabolic process | 25 | 1.64E-07 | 3.74E-05 |  |
| organophosphate metabolic process | 14 | 9.14E-05 | 7.46E-03 |  |
| purine-containing compound metabolic process | 8 | 3.48E-04 | 2.14E-02 |  |
| ribonucleotide metabolic process | 8 | 2.35E-04 | 1.54E-02 |  |
| ribose phosphate metabolic process | 8 | 2.93E-04 | 1.86E-02 |  |
| purine ribonucleoside diphosphate metabolic process | 7 | 2.55E-08 | 6.79E-06 |  |
| purine nucleoside diphosphate metabolic process | 7 | 2.55E-08 | 6.67E-06 |  |
| nucleoside diphosphate metabolic process | 8 | 2.32E-08 | 6.41E-06 |  |
| ribonucleoside diphosphate metabolic process | 7 | 4.14E-08 | 1.02E-05 |  |
| ATP metabolic process | 8 | 3.24E-05 | 3.21E-03 |  |
| generation of precursor metabolites and energy | 9 | 1.13E-04 | 8.92E-03 |  |
| nucleoside diphosphate phosphorylation | 8 | 3.11E-09 | 1.14E-06 |  |
| nucleotide phosphorylation | 8 | 3.50E-09 | 1.20E-06 |  |
| pyruvate metabolic process | 8 | 1.65E-09 | 6.96E-07 |  |
| monocarboxylic acid metabolic process | 12 | 3.59E-06 | 4.74E-04 |  |
| carboxylic acid metabolic process | 15 | 9.12E-06 | 1.04E-03 |  |
| oxoacid metabolic process | 16 | 2.35E-06 | 3.37E-04 |  |
| organic acid metabolic process | 18 | 1.54E-07 | 3.56E-05 |  |
| cholesterol efflux | 4 | 9.75E-06 | 1.10E-03 | lipid metabolism |
| innate immune response in mucosa | 3 | 1.69E-04 | 1.24E-02 | immune system |
| mucosal immune response | 3 | 3.75E-04 | 2.28E-02 | immune system |
| organ or tissue specific immune response | 3 | 5.21E-04 | 3.03E-02 | immune system |
| antibacterial humoral response | 6 | 7.68E-08 | 1.81E-05 | immune system |
| antimicrobial humoral response | 10 | 1.23E-10 | 6.29E-08 | immune system |
| defense response to bacterium | 15 | 6.80E-11 | 3.62E-08 | immune system |
| response to bacterium | 21 | 1.58E-12 | 1.03E-09 | immune system |
| hydrogen peroxide catabolic process | 5 | 1.27E-06 | 2.05E-04 |  |
| hydrogen peroxide metabolic process | 5 | 5.10E-06 | 6.43E-04 |  |
| reactive oxygen species metabolic process | 5 | 2.32E-04 | 1.53E-02 |  |
| killing of cells of other organism | 4 | 1.80E-05 | 1.96E-03 |  |
| cell killing | 7 | 1.51E-08 | 4.35E-06 |  |
| triglyceride homeostasis | 4 | 1.80E-05 | 1.95E-03 | lipid metabolism |
| acylglycerol homeostasis | 4 | 1.80E-05 | 1.93E-03 | lipid metabolism |
| lipid homeostasis | 6 | 9.08E-05 | 7.50E-03 | lipid metabolism |
| chemical homeostasis | 16 | 4.08E-05 | 3.75E-03 |  |
| homeostatic process | 20 | 1.92E-05 | 2.00E-03 |  |
| regulation of biological quality | 38 | 9.91E-07 | 1.72E-04 |  |
| glucose 6-phosphate metabolic process | 3 | 2.26E-04 | 1.50E-02 | carbohydrate metabolism |
| defense response to fungus | 5 | 1.93E-06 | 2.92E-04 |  |
| response to fungus | 5 | 4.56E-06 | 5.91E-04 |  |
| positive regulation of fatty acid biosynthetic process | 3 | 2.95E-04 | 1.87E-02 | lipid metabolism |
| positive regulation of small molecule metabolic process | 5 | 1.83E-04 | 1.26E-02 |  |
| positive regulation of lipid biosynthetic process | 5 | 3.52E-05 | 3.35E-03 | lipid metabolism |
| regulation of lipid biosynthetic process | 6 | 1.07E-04 | 8.54E-03 | lipid metabolism |
| gluconeogenesis | 3 | 2.95E-04 | 1.86E-02 | carbohydrate metabolism |
| hexose biosynthetic process | 3 | 3.75E-04 | 2.29E-02 | carbohydrate metabolism |
| hexose metabolic process | 7 | 3.45E-06 | 4.63E-04 | carbohydrate metabolism |
| monosaccharide metabolic process | 8 | 7.32E-07 | 1.33E-04 | carbohydrate metabolism |
| monosaccharide biosynthetic process | 4 | 3.05E-05 | 3.04E-03 | carbohydrate metabolism |
| small molecule biosynthetic process | 11 | 7.52E-06 | 8.93E-04 |  |
| carbohydrate biosynthetic process | 5 | 1.74E-04 | 1.23E-02 | carbohydrate metabolism |
| glucose metabolic process | 5 | 6.42E-05 | 5.63E-03 | carbohydrate metabolism |
| response to estradiol | 5 | 3.22E-06 | 4.41E-04 |  |
| complement activation, classical pathway | 6 | 3.86E-07 | 8.05E-05 | immune system |
| humoral immune response mediated by circulating immunoglobulin | 6 | 6.73E-07 | 1.26E-04 | immune system |
| immunoglobulin mediated immune response | 7 | 2.42E-06 | 3.44E-04 | immune system |
| B cell mediated immunity | 7 | 2.57E-06 | 3.59E-04 | immune system |
| adaptive immune response based on somatic recombination of immune receptors built from immunoglobulin superfamily domains | 7 | 2.72E-05 | 2.73E-03 | immune system |
| adaptive immune response | 8 | 6.27E-05 | 5.56E-03 | immune system |
| lymphocyte mediated immunity | 8 | 1.14E-06 | 1.88E-04 | immune system |
| leukocyte mediated immunity | 8 | 4.97E-06 | 6.32E-04 | immune system |
| regulation of complement activation | 3 | 3.75E-04 | 2.26E-02 | immune system |
| regulation of immune effector process | 7 | 9.57E-04 | 4.89E-02 | immune system |
| platelet aggregation | 3 | 4.21E-04 | 2.52E-02 |  |
| platelet activation | 4 | 1.73E-04 | 1.22E-02 |  |
| blood coagulation | 6 | 3.84E-05 | 3.59E-03 |  |
| coagulation | 6 | 3.84E-05 | 3.61E-03 |  |
| hemostasis | 6 | 4.04E-05 | 3.74E-03 |  |
| regulation of body fluid levels | 9 | 1.72E-05 | 1.89E-03 |  |
| wound healing | 7 | 1.57E-04 | 1.17E-02 |  |
| response to wounding | 7 | 5.70E-04 | 3.26E-02 |  |
| lipid storage | 5 | 5.10E-06 | 6.37E-04 | lipid metabolism |
| maintenance of location | 6 | 2.03E-04 | 1.37E-02 |  |
| modulation by host of viral process | 3 | 4.69E-04 | 2.78E-02 | immune system |
| biological process involved in interaction with symbiont | 5 | 5.66E-05 | 5.08E-03 |  |
| biological process involved in symbiotic interaction | 6 | 1.46E-04 | 1.12E-02 |  |
| antimicrobial humoral immune response mediated by antimicrobial peptide | 6 | 9.17E-07 | 1.63E-04 | immune system |
| regulation of triglyceride metabolic process | 4 | 7.30E-05 | 6.13E-03 | lipid metabolism |
| positive regulation of cholesterol transport | 3 | 6.36E-04 | 3.59E-02 | lipid metabolism |
| regulation of cholesterol transport | 4 | 1.73E-04 | 1.24E-02 | lipid metabolism |
| regulation of sterol transport | 4 | 1.73E-04 | 1.23E-02 | lipid metabolism |
| positive regulation of sterol transport | 3 | 6.36E-04 | 3.57E-02 | lipid metabolism |
| regulation of blood coagulation | 6 | 1.12E-06 | 1.86E-04 |  |
| regulation of coagulation | 6 | 1.62E-06 | 2.50E-04 |  |
| regulation of multicellular organismal process | 35 | 5.56E-10 | 2.66E-07 |  |
| regulation of response to external stimulus | 16 | 1.11E-06 | 1.88E-04 |  |
| regulation of wound healing | 8 | 1.72E-07 | 3.87E-05 |  |
| regulation of response to wounding | 8 | 6.92E-07 | 1.27E-04 |  |
| regulation of response to stress | 19 | 1.05E-06 | 1.80E-04 |  |
| regulation of hemostasis | 6 | 1.35E-06 | 2.11E-04 |  |
| cholesterol biosynthetic process | 3 | 6.99E-04 | 3.85E-02 | lipid metabolism |
| sterol biosynthetic process | 3 | 9.12E-04 | 4.70E-02 | lipid metabolism |
| sterol metabolic process | 5 | 2.02E-04 | 1.37E-02 | lipid metabolism |
| cholesterol metabolic process | 5 | 1.42E-04 | 1.10E-02 | lipid metabolism |
| secondary alcohol metabolic process | 5 | 2.11E-04 | 1.42E-02 |  |
| secondary alcohol biosynthetic process | 3 | 6.99E-04 | 3.87E-02 |  |
| vitamin transport | 4 | 9.66E-05 | 7.84E-03 |  |
| cellular oxidant detoxification | 9 | 8.59E-09 | 2.68E-06 |  |
| cellular detoxification | 10 | 1.33E-09 | 5.79E-07 |  |
| detoxification | 10 | 4.75E-09 | 1.59E-06 |  |
| response to toxic substance | 10 | 4.10E-08 | 1.03E-05 |  |
| cellular response to toxic substance | 10 | 1.97E-09 | 7.66E-07 |  |
| cellular response to chemical stimulus | 26 | 6.82E-05 | 5.87E-03 |  |
| cholesterol homeostasis | 6 | 3.45E-06 | 4.68E-04 | lipid metabolism |
| sterol homeostasis | 6 | 3.73E-06 | 4.87E-04 | lipid metabolism |
| intermembrane lipid transfer | 4 | 1.73E-04 | 1.23E-02 | lipid metabolism |
| negative regulation of endopeptidase activity | 22 | 3.87E-19 | 2.78E-15 | protein metabolism |
| regulation of endopeptidase activity | 23 | 9.80E-17 | 1.17E-13 | protein metabolism |
| regulation of peptidase activity | 24 | 4.09E-17 | 6.53E-14 | protein metabolism |
| regulation of hydrolase activity | 28 | 8.51E-14 | 6.44E-11 |  |
| regulation of catalytic activity | 37 | 3.36E-11 | 1.93E-08 |  |
| regulation of molecular function | 39 | 1.94E-09 | 7.76E-07 |  |
| negative regulation of peptidase activity | 22 | 4.84E-19 | 2.32E-15 |  |
| negative regulation of proteolysis | 22 | 4.73E-17 | 6.80E-14 |  |
| negative regulation of cellular protein metabolic process | 24 | 4.49E-11 | 2.48E-08 |  |
| negative regulation of protein metabolic process | 25 | 2.20E-11 | 1.32E-08 |  |
| negative regulation of macromolecule metabolic process | 33 | 3.00E-07 | 6.34E-05 |  |
| negative regulation of metabolic process | 34 | 4.09E-07 | 8.15E-05 |  |
| negative regulation of biological process | 47 | 1.73E-06 | 2.64E-04 |  |
| negative regulation of nitrogen compound metabolic process | 28 | 5.24E-06 | 6.44E-04 |  |
| negative regulation of cellular metabolic process | 28 | 1.88E-05 | 1.98E-03 |  |
| negative regulation of cellular process | 44 | 2.58E-06 | 3.56E-04 |  |
| negative regulation of hydrolase activity | 24 | 9.04E-19 | 3.25E-15 |  |
| negative regulation of catalytic activity | 27 | 1.05E-15 | 9.45E-13 |  |
| negative regulation of molecular function | 28 | 2.17E-13 | 1.56E-10 |  |
| iron ion transport | 5 | 3.52E-05 | 3.38E-03 |  |
| transition metal ion transport | 6 | 3.66E-05 | 3.46E-03 |  |
| phagocytosis, engulfment | 4 | 3.46E-04 | 2.13E-02 | immune system |
| phagocytosis | 6 | 1.63E-04 | 1.21E-02 | immune system |
| vesicle-mediated transport | 16 | 4.60E-04 | 2.73E-02 |  |
| plasma membrane invagination | 4 | 5.54E-04 | 3.20E-02 |  |
| membrane invagination | 4 | 7.59E-04 | 4.13E-02 |  |
| endocytosis | 9 | 3.53E-04 | 2.16E-02 |  |
| regulation of receptor-mediated endocytosis | 4 | 5.24E-04 | 3.04E-02 |  |
| regulation of endocytosis | 5 | 8.77E-04 | 4.55E-02 |  |
| regulation of cellular component organization | 22 | 8.17E-04 | 4.31E-02 |  |
| regulation of vesicle-mediated transport | 14 | 5.23E-08 | 1.28E-05 |  |
| positive regulation of endocytosis | 4 | 5.54E-04 | 3.18E-02 |  |
| negative regulation of immune effector process | 5 | 1.21E-04 | 9.53E-03 | immune system |
| regulation of interleukin-1 production | 4 | 6.17E-04 | 3.49E-02 | immune system |
| regulation of cytokine production | 14 | 2.02E-06 | 3.03E-04 | immune system |
| cellular iron ion homeostasis | 4 | 7.98E-04 | 4.31E-02 |  |
| iron ion homeostasis | 5 | 1.50E-04 | 1.14E-02 |  |
| transition metal ion homeostasis | 5 | 8.20E-04 | 4.31E-02 |  |
| immunoglobulin production | 6 | 5.37E-05 | 4.88E-03 | immune system |
| production of molecular mediator of immune response | 6 | 6.44E-05 | 5.61E-03 | immune system |
| regulation of cell shape | 6 | 1.07E-04 | 8.49E-03 |  |
| regulation of cell morphogenesis | 8 | 6.27E-05 | 5.53E-03 |  |
| regulation of anatomical structure morphogenesis | 15 | 7.10E-06 | 8.50E-04 |  |
| regulation of developmental process | 22 | 8.17E-04 | 4.33E-02 |  |
| negative regulation of supramolecular fiber organization | 6 | 1.63E-04 | 1.20E-02 |  |
| regulation of supramolecular fiber organization | 9 | 1.26E-04 | 9.79E-03 |  |
| regulation of inflammatory response | 9 | 8.46E-06 | 9.88E-04 | immune system |
| regulation of defense response | 11 | 1.50E-05 | 1.66E-03 | immune system |
| regulation of ERK1 and ERK2 cascade | 7 | 2.52E-04 | 1.64E-02 |  |
| negative regulation of cytokine production | 6 | 7.31E-04 | 4.01E-02 |  |
| negative regulation of multicellular organismal process | 15 | 1.02E-05 | 1.14E-03 |  |
| negative regulation of response to external stimulus | 8 | 1.91E-04 | 1.31E-02 |  |
| positive regulation of cytokine production | 8 | 7.47E-04 | 4.08E-02 |  |
| positive regulation of multicellular organismal process | 21 | 4.07E-07 | 8.25E-05 |  |
| positive regulation of intracellular signal transduction | 13 | 3.02E-04 | 1.90E-02 |  |
| positive regulation of signal transduction | 16 | 8.89E-04 | 4.59E-02 |  |
| positive regulation of cell communication | 17 | 8.52E-04 | 4.45E-02 |  |
| regulation of multicellular organismal development | 15 | 4.76E-04 | 2.79E-02 |  |
| unclassified | 8 | 2.55E-04 | 1.65E-02 |  |
| regulation of transcription by RNA polymerase II | 2 | 6.49E-04 | 3.62E-02 |  |
